# Regional variations in multimorbidity burden among office-based physicians in Germany

**DOI:** 10.1093/eurpub/ckad039

**Published:** 2023-03-15

**Authors:** Isabel Geiger, Ronja Flemming, Wiebke Schüttig, Leonie Sundmacher

**Affiliations:** Institute for Medical Information Processing, Biometry, and Epidemiology, Ludwig-Maximilians-University, Munich, Germany; Pettenkofer School of Public Health, Munich, Germany; Chair of Health Economics, Technical University of Munich, Germany; Chair of Health Economics, Technical University of Munich, Germany; Chair of Health Economics, Technical University of Munich, Germany

## Abstract

**Background:**

Multimorbidity is associated with higher utilization of healthcare services. However, many countries do not consider multimorbidity when estimating physician supply. The main aim of this study was to assess how regional multimorbidity levels can be integrated when estimating the need for office-based physician supply.

**Methods:**

Claims data were used to measure and compare the proportions of multimorbid patients of GPs, ophthalmologists, orthopaedic specialists and neurologists, and examine spatial variations through Bernoulli cluster analysis of regional multimorbidity levels. To explore the interrelationship between current capacities and spatial occurrence of high-rate clusters, clusters were compared with the current supply of physicians.

**Results:**

About 17 239 488 individuals out of approximately 67 million records were classified as multimorbid. Multimorbidity levels varied greatly between physician disciplines (31.5–60.1%). Bernoulli cluster analysis demonstrated that many high-rate areas were found for all specialized physicians, but clusters varied partially by size and location. The comparison with current physician supply at cluster level showed that more than a third of clusters with a significantly higher share of morbid patients seeing a GP are met, on an average, by GP supply below targeted values. In turn, clusters with significantly higher multimorbidity levels of specialized physicians were met, on an average, by supply that exceeded targeted values.

**Conclusion:**

Our study offers an approach to how to include discipline-specific multimorbidity at area level when estimating physician supply and discusses its relevance. The outcomes of our article can be used by policymakers to advance current planning strategies and to improve the quality of office-based care.

## Introduction

Demographic changes, specifically the ageing of the population, and the increase in non-communicable diseases are associated with an increasing number of people suffering from co-occurring diseases.^1^ The prevalence of multiple chronic conditions has been observed to rise with age, especially for people aged 50 years and above, over the last two decades.^2^ However, not only older adults are affected by the rise in multiple non-communicable diseases, but a large percentage of young adults also carry the burden of several chronic diseases with respective consequences on health outcomes.^3^^,^^4^

Conventionally, if several chronic conditions are present in a person simultaneously, he or she is classified as multimorbid.^5^ Previous research suggests a strong correlation between healthcare utilization (e.g. outpatient visits, prescriptions) and patients suffering from several chronic conditions.^6–9^ The literature on older adults, for instance, shows that multimorbid patients see physicians more than twice as often as patients with only one chronic condition.^10^ Additionally, polypharmacy and mental health difficulties are commonly present in multimorbid patients, thus intensifying the need for extended consultation lengths. However, the precise effect on the consultation length remains unknown.^7^^,^^11^ Research in young adults additionally shows that multimorbidity is related to productivity loss, which further increases the economic burden attributed to multimorbidity.^3^

Previous multimorbidity studies have focused on the potential influence on and occurrence in primary care in general, irrespective of the physicians’ speciality and regional manifestations.^10^^,^^12^^,^^13^ Thus, it remains mostly unclear whether the share of multimorbid people at area level affects all office-based physicians similarly or if some physician disciplines are more affected in certain areas than others. This is particularly interesting in countries such as Germany where the role of GPs as care coordinators and ‘gatekeepers’ is less dominant.^14^ As multimorbidity is associated with an increased need for care and care coordination, this information is essential for needs-based physician planning in order to allocate resources effectively and incentivise coordinated and integrated care.^15^

In Germany, the number of physicians needed per discipline and planning level are designated by the self-administered German National Association of Statutory Health Insurance Physicians (KBV) through respective physician-to-population ratios, which are corrected by a demographic factor. If needed, regional entities (regional associations of the statutory health insurance physicians) can further adjust these ratios locally to so-called adapted physician-to-population ratios (‘angepasste Verhältniszahlen’ [acronym AVZ]). The AVZ is considered to account for the population’s regional need for healthcare (based on the possibility of accounting for regional characteristics) and is thus primarily used as a measure for workforce planning. However, multimorbidity levels are not incorporated in the AVZ.^15^

The main objective of this study is to assess whether regional multimorbidity levels should be incorporated when estimating the need for office-based physician supply, exemplified by four selected physician disciplines. The research addresses the questions as to how claims data can be used to classify multimorbidity levels and whether multimorbidity should be measured for every physician discipline individually. Additionally, we examine whether contemporary supply of physicians can meet the potentially greater need for care and care coordination in high-rate areas by comparing current physician supply with high-rate clusters.

## Methods

### Data source and extraction

The primary data source for our cross-sectional study was claims data from the KBV, which covers all publicly insured Germans (approximately 89% of the German population). Our dataset was recorded in 2015 and includes data from all office-based physician visits irrespective of which public sickness fund an individual belongs to.

Multimorbidity is defined following the disease categories recommended by Barnett et al.,^5^ which cover 40 chronic disease groups, using the disease count approach. ICD-10 German Modification (GM) codes were added to the disease categories and validated through medical experts. A full list of ICD-10-GM codes used in this article can be found in Supplementary file S1. In line with previous German studies,^12^^,^^16^ a person was classified as multimorbid if he or she suffered from three or more recorded chronic diseases simultaneously. To account for coding errors, a person had to be diagnosed with an ICD-10-GM code from the same chronic disease group in two different quarters of the year 2015. The number of multimorbid patients was aggregated at district area level corresponding to the respective planning level, so-called ‘Mittelbereiche’ [acronym MB] for general practitioners (GPs) and ‘Kreisregionen’ [acronym KR] for other disciplines.

The range of patient contacts per year differed greatly between disciplines, so we selected disciplines with the highest outpatient visit rates: GPs and ophthalmologists.^13^ Additionally, we included neurologists and orthopaedic specialists because, after cancer and cardiovascular diseases, musculoskeletal and neurological diseases account for the biggest share of all chronic diseases in Germany.^17^ Next to the number of multimorbid patients, the number of patients who visited the respective physician discipline in 2015 per area was also available.

The number of physicians per discipline and AVZs were obtained at regional level from a 2016 KBV internal survey. All data were prepared using the open-source programme R (version 4.0.3).

### Statistical analysis

First, we measured multimorbidity levels based on claims data following the disease count approach (see above) and descriptively summarized multimorbidity shares in both absolute numbers and proportions per physician discipline to assess differences in multimorbidity levels. Boxplots are provided as measures of overall variation between disciplines. Great variation in multimorbidity levels would substantiate incorporating multimorbidity levels individually when estimating physician supply.

Second, to evaluate the regional variation in multimorbidity between physician disciplines and to identify spatial clusters, we performed a spatial Bernoulli cluster analysis. The spatial unit to detect clusters with a significantly higher or lower proportions of multimorbid patients represents MBs and KRs for GPs and all specialized physicians, respectively. Based on Kulldorff (1996), *N* denotes the spatial point process with *N*(*A*) being a random number of points in the set *A* ⊂ *G*, with *G* being the geographical space. When the window moves over the study area it defines a collection Z of zones *Z* ⊂ *G*. Within *Z* ⊂ *G*, each individual has the probability (*p*) of being multimorbid. Outside *Z*, the probability of being multimorbid is *q*. The likelihood function can be written as^18^: where *n*_Z_ is the number of multimorbid patients in *Z* and *n*_G_ the total number of multimorbid patients in the study area.^18^


$$L\left(Z,p,q\right)=p^{n_{Z}}{\left(1-p\right)}^{\mu \left(Z\right)-n_{Z}}{q^{n_{G}-n_{Z}}(1-q)}^{\left(\mu \left(G\right)-\mu \left(Z\right)\right)-(n_{G}-n_{Z})}$$

The maximum size of a spatial cluster in the at-risk population was set to 50% and the window was a circular shape. No additional modifications were made. Every analysis included 999 Monte Carlo replications to obtain the likelihood ratios and the corresponding *P* values. Statistical significance was set at a *P* values of <0.01. The Bernoulli model is used to detect proportionally higher rates of expected multimorbid patients under the assumption that the KBV data of 2015 are representative of the German publicly insured population. High-rate clusters are hypothesized to point to a higher burden of multimorbidity in the respective physician discipline, which may lead to a greater need for health services and integrated care approaches in that area. Low-rate clusters of multimorbid patients are assumed to indicate no additional need for health services. Clusters are summarized descriptively and compared between physician disciplines. Cluster detection is performed using SaTScan (version 9.6). To test the robustness of the results, proportions of multimorbid patients were also analyzed for high- and low-rate clusters using spatial autocorrelation mapping through Local Moran’s *I* in QGIS (version 3.22.4), which can be expressed as^19^:


$$I_{i}=z_{i}\sum_{j}w_{\mathrm{ij}}z_{j}$$

Third, to assess whether contemporary supply of physicians can meet the potentially greater need for care and care coordination in high-rate areas, we compared high-rate clusters with current supply (graphically and descriptively). The underlying hypothesis was that regions with a greater likelihood of multimorbid patients require a high(er) number of physicians to meet their care needs. As measures for physician capacities, we derived degrees of supply coverage per area by comparing the actual number of physicians with the targeted number of physicians using the AVZ as a basis for calculation of the targeted numbers. The categories for supply coverage were adapted from KBV classifications for physician planning purposes: Level 1 ‘shortage’ refers to an available supply of physicians compared with the computed number of physicians needed in a planning unit of less than 75% for GPs and less than 50% for specialists. Level 2 ‘imminent shortage’ refers to a care coverage of 75–99% for GPs and 50–99% for specialists. Level 3 ‘target coverage’ is located between 100% and 109% care coverage for all office-based physicians. Level 4 ‘potential excess’ refers to a care coverage of 110–139% and lastly, level 5 ‘excess’ refers to a care coverage of 140% and above. As the AVZ should already incorporate local need requirements and the lack of alternative classifications, levels 3–5 are considered acceptable to meet the needs of high-rate areas.

All maps for the results section were generated using QGIS (version 3.22.4).

## Results

### Descriptive summary

In total, claims data from 67 233 964 patients out of approximately 70.8 million publicly insured individuals in the year 2015 were recorded in the dataset. Verified ICD-10-GM codes from claims data, which were assigned to 40 disease categories, were used to identify multimorbid patients individually for every physician discipline across Germany. Although GPs show the greatest absolute number of multimorbid patients (*n* = 17 239 488), neurologists have the greatest relative number of multimorbid patients (60.1%) (table 1). GPs indicate the lowest proportion of multimorbid patients with 31.5%. Overall, ophthalmologists have the highest average case rate per year (*n* = 5145).

**Table 1:** Descriptive summary of selected office-based physicians and their treated patients in Germany in 2015

Physician discipline	Unit	Number of physicians	Patients	Average number of cases per year	Multimorbid patients	Share of multimorbid patients (%)
GPs	MB	52 527	54 799 570	3940	17 239 488	31.5
Neurologists	KR	4683	4 386 298	2204	2 637 461	60.1
Ophthalmologists	KR	5434	16 195 148	5145	7 145 558	44.1
Orthopaedic specialists	KR	5483	11 659 090	3824	4 722 933	40.5

**Figure 1:**
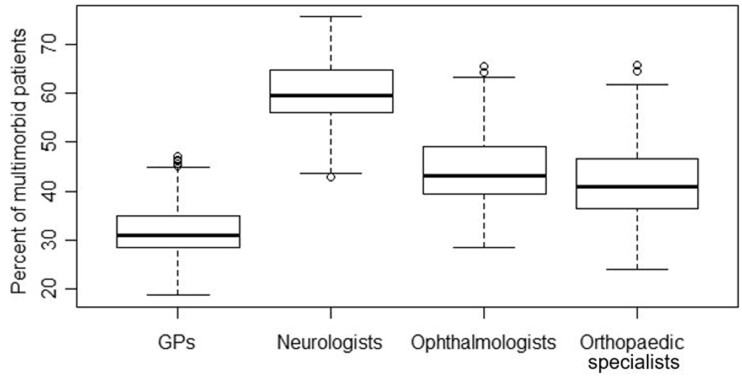
Boxplots of multimorbidity shares per selected office-based physician disciplines in Germany in 2015. NB: multimorbidity shares of GPs are measured in MB; all other disciplines in KR.

In figure 1, boxplots using percentages of multimorbid patients per discipline at the respective planning level show that neurologists (media*n* = 59.5%) have the highest share of multimorbid patients and GPs the lowest (median = 30.9%). Ophthalmologists and orthopaedic specialists show a similar level of multimorbidity with medians of 43.0% and 40.8%, respectively.

### Regional multimorbidity

**Figure 2:**
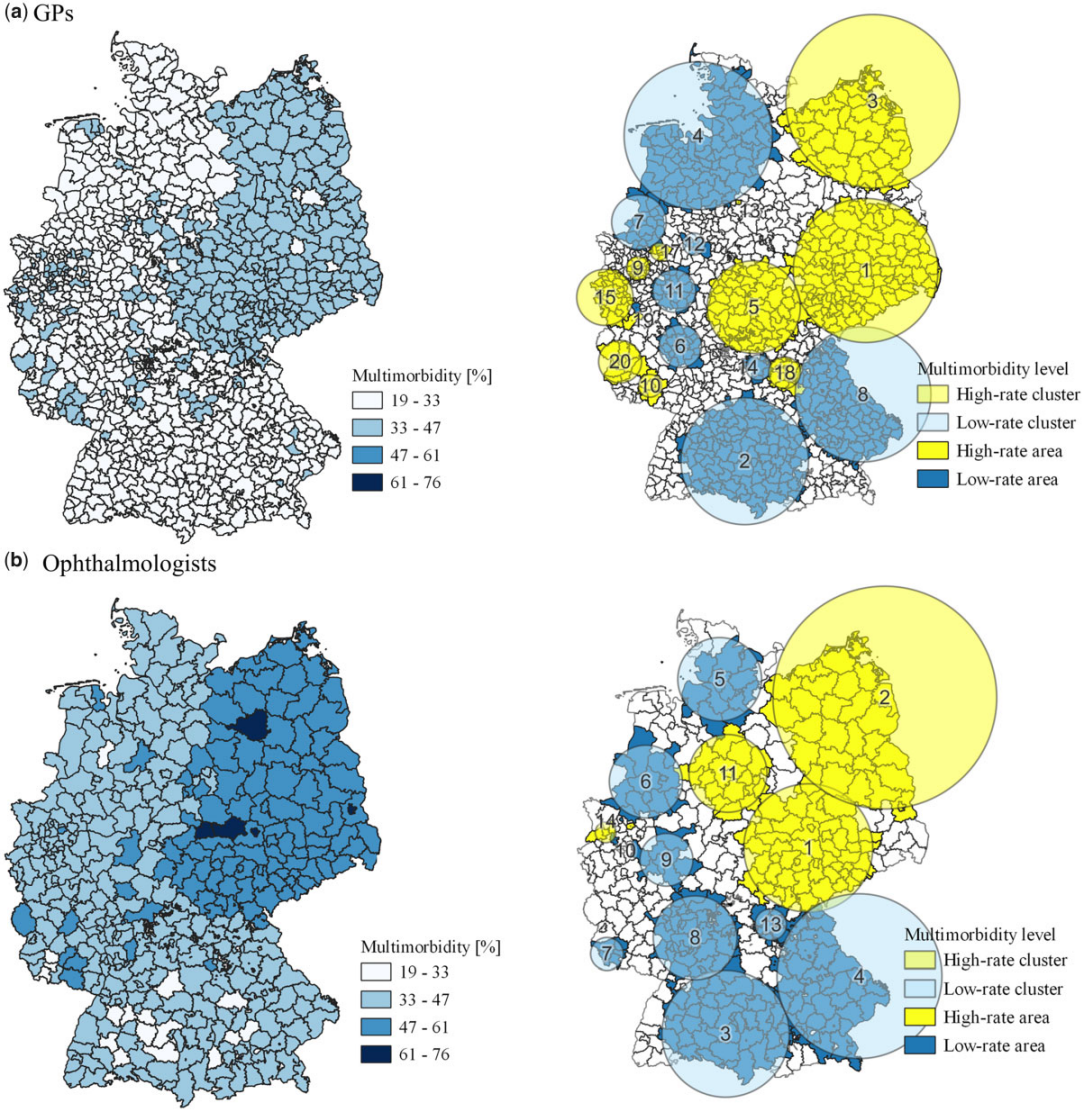
Multimorbidity levels in percentages (left-hand side) and identified clusters (right-hand side) of GPs (a) and ophthalmologists (b) in Germany in 2015. All maps are displayed at the respective planning level.

Figure 2 illustrates multimorbidity levels in percentages at the planning level, respective high-rate clusters and underlying areas of all physician disciplines. Clusters were numbered consecutively using Arabic numbers based on the likelihood ratio starting with the cluster showing the highest likelihood ratio. For example, C-1 represents the most likely cluster in the analysis.

**Figure 3:**
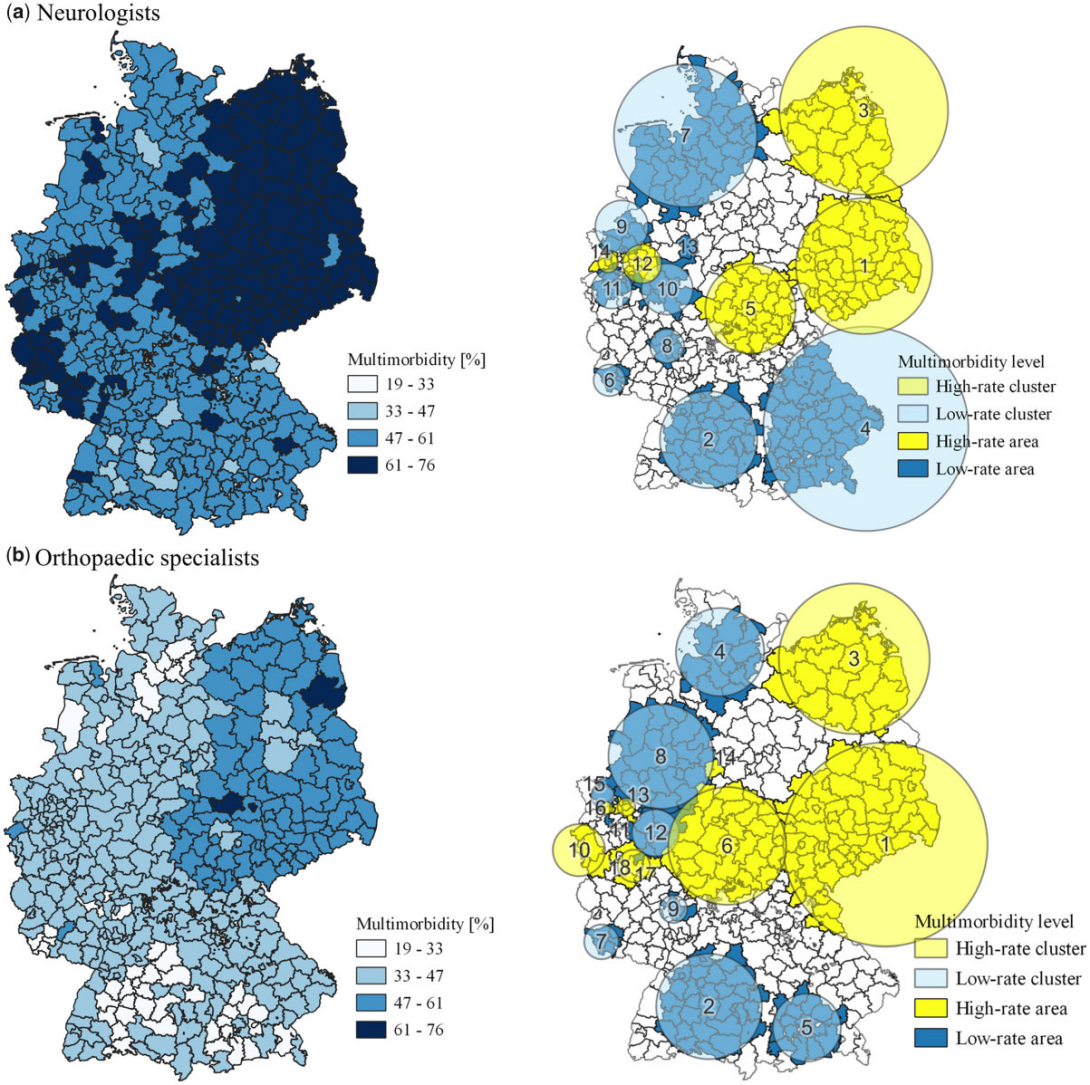
Multimorbidity levels in percentages (left-hand side) and identified clusters (right-hand side) of neurologists (a) and orthopaedic specialist (b) in Germany in 2015. All maps are displayed at the respective planning level.

As illustrated in figures 2 and 3, the spatial scan resulted in 20 clusters for GPs divided into 11 high-rate clusters covering 292 MBs and nine low-rate clusters covering 324 MBs. Five out of 14 clusters detected for both ophthalmologists and neurologists covering 101 KRs and 97 KRs, respectively, were found as high-rate clusters with the remaining nine low-rate clusters covering 153 KRs and 146 KRs. Orthopaedic specialists showed 18 clusters with eight high-rate and ten low-rate, covering 122 KRs and 107 KRs, respectively. The relative risk within a cluster varied between 0.73 and 1.35 depending on the cluster and physician discipline. The most likely high-rate cluster (C-1) with the highest relative risk in all disciplines was found in the east of Germany extending over Bavaria, Brandenburg, Saxony, Saxony-Anhalt and Thuringia. The cluster varies in size with a radius between 128 km (neurologists) and 177 km (orthopaedic specialists). A detailed overview of the scanning results including log likelihood ratios, *P* values, location and radius of each cluster, to name but a few, can be found in Supplementary file S2.

As MBs and KRs are different planning units, we were unable to directly compare the results of GPs with the other physician disciplines. However, figures 2 and 3 illustrate that GPs show similar but not identical clusters as compared with neurologists and other physician disciplines. Nonetheless, the following assessment is restricted to the comparison of neurologists, ophthalmologists and orthopaedic specialists.

In total, 159 out of 385 KRs were found to be high-rate areas through the Bernoulli analysis. Of these areas, 39.6% were found in all three physician disciplines, 22.0% in two disciplines and the remaining 38.4% in only one discipline. Overall, 98 KRs (61.6%) are shared by at least two physician disciplines, with orthopaedic specialists sharing most high-rate areas (*n* = 94) with both ophthalmologists and neurologists, followed by neurologists sharing 86 KRs and ophthalmologists sharing 79 KRs. In other words, neurologists share 88.7% of their high-rate areas with at least one physician discipline, ophthalmologists share 78.2% and orthopaedic specialists 77.0%. Overlapping areas were found in 130 KRs out of 198 low-rate areas (65.7%) for all physician disciplines, with orthopaedic specialists sharing 92.5%, neurologists 82.9% and ophthalmologists 77.1% with at least one other physician discipline.

At a cluster level, one high-rate cluster was found with identical size and location for both neurologists (C-14) and ophthalmologists (C-14). Additionally, one identical low-rate cluster was found for ophthalmologists (C-7, C-10), orthopaedic specialists (C-7) and neurologists (C-6) as well as two between ophthalmologists (C-5, C-10) and orthopaedic specialists (C-4, C-11). Moreover, one identical low-rate cluster was shared by ophthalmologists (C-5) and orthopaedic specialists (C-4) and one low-rate cluster between orthopaedic specialists (C-2) and neurologists (C-2). Overall, eight high-rate clusters (C-1, C-3, C-6, C-10, C-13, C-14, C-16, C-17) and six low-rate clusters (C-5, C-8, C-9, C-12, C-15, C-18) of orthopaedic specialists, four high-rate clusters (C-1, C-2, C-11, C-12) and five low-rate clusters (C-3, C-4, C-6, C-8, C-13) of ophthalmologists and four high-rate clusters (C-1, C-3, C-5, C-12) and six low-rate clusters (C-4, C-7, C-8, C-9, C-11, C-13) of neurologists did not (fully) match any cluster from another discipline (figures 2 and 3).

The robustness test with Moran’s *I* resulted in 50.0% confirmed high-rate and 42.6% low-rate areas for GPs, 70.3% confirmed high-rate and 43.1% low-rate areas for ophthalmologists, 64.9% confirmed high-rate and 32.9% low-rate areas for neurologists as well as 59.0% confirmed high-rate and 57.0% low-rate areas for orthopaedic specialists. However, only 16.1% of high-rate and 17.0% of low-rate areas of GPs, 14.5% of high-rate and 2.9% of low-rate areas of ophthalmologists, 16.0% of high-rate and 9.4% of low-rate areas of neurologists and 12.2% of high-rate and 9.0% of low-rate areas identified by Moran’s *I* were not detected by the Bernoulli model. A detailed overview of the test results is provided in Supplementary files S3 and S4 with corresponding maps provided in Supplementary file S5.

### Comparison with current capacities

As high-rate areas are derived from clustered regions, we compared the average supply levels at cluster level. The results in figure 3 show that, in the case of GPs, average supply in five high-rate clusters (C-1, C-3, C-13, C-15, C-20) fall below the targeted coverage, one high-rate cluster is met by target supply (C-13), and average supply exceeds targeted values in five clusters (C-5, C-9, C-10, C-17, C-18). In contrast to GPs, all high-rate clusters of ophthalmologists, neurologists and orthopaedic specialists are met by an average physician supply above targeted coverage. A detailed overview of all clusters, respective planning levels and corresponding physician supply can be found in Supplementary files S3 and S4 with corresponding maps provided in Supplementary file S6.

## Discussion

The objective of this article was to assess whether regional multimorbidity levels should be integrated when estimating the need for office-based physician supply, based on four selected physician disciplines in Germany. A disease count approach using ICD-10-GM codes from claims data was applied to identify multimorbid patients for every physician discipline at area level. Although multimorbidity shares vary between physician disciplines overall, the spatial Bernoulli cluster analysis highlighted that many high-rate and even more low-rate areas were found to overlap between specialized physicians, only varying partially in cluster size and location. By comparing current physician supply at cluster level, we demonstrated that care provision in more than a third of the high-rate clusters of GPs falls, on an average, below the targeted physician supply. In turn, all high-rate clusters of specialized physicians were met by an average supply that exceeded targeted values.

To our knowledge, this study is the first to use nationwide claims data without age restriction to identify and compare physician-specific multimorbidity shares at area level. Previous research on multimorbidity primarily relied on data gathered from telephone interviews^20–22^ or data from single sickness funds,^12^^,^^16^^,^^23^^,^^24^ and was thus spatially limited and/or unspecific regarding physician disciplines. With our dataset, we were able to provide an overview of multimorbidity levels across Germany for health resource planning with a high resolution (KRs or MBs) and differentiated by physician discipline using ICD-10-GM codes, which can be applied for future planning approaches.

Our results confirm that the shares of multimorbid patients vary significantly between physician disciplines, but the locations of high- and low-rate areas derived by cluster analysis frequently overlap. Nonetheless, even physician disciplines that show similar overall multimorbidity shares, such as ophthalmologists and orthopaedic specialists, may differ in their spatial distribution of high-rate clusters of multimorbid patients. The robustness test additionally shows that not all detected areas could be validated but also highlights spatial differences in high- and low-rate areas between physicians, which may be considered when estimating needs-based supply, if proven robust to supply influences. One major challenge in this regard, however, remains concerning the translation of these increased care needs into physician requirements. Sundmacher et al.,^15^ for instance, used multimorbidity as an explanatory variable in their regression-based model when calculating physician requirements in points, which were used as a proxy for physician time. More evidence is needed to translate multimorbidity directly into physician capacities to improve workforce planning.

The comparison of high-rate clusters of multimorbid patients with specialized physician supply demonstrated average supply that exceeded targeted values. However, no clear pattern was visible regarding physician capacities in high-rate clusters compared with supply outside of these areas. Incorporating multimorbidity levels into German workforce planning might change current AVZs and thus, change the current care coverage in high-rate clusters to supply that does not exceed targeted values.

Looking at GP capacities, the imminent shortage in Germany specifically in high-rate areas with assumed higher need for health services and integrated care is highlighted. Although GPs presented the lowest overall level of multimorbid patients, they provide care for the second highest number of cases per year and see the highest absolute number of multimorbid patients, which further underlines their role as care coordinators. Thus, to reduce the workload on physicians and to improve integrated care, recent studies tested innovative methods of healthcare delivery for people with multimorbidity and chronic diseases.^25–27^ Using the evidence provided in this article combined with recent studies,^28^^,^^29^ we recommend healthcare planners to further target the existing shortage of GPs and specifically consider regions with a greater likelihood of multimorbid patients next to other drivers such as age and socio-economic status (SES) to ensure continuous healthcare provision in such regions.

All analyses for this article were done with absolute numbers or percentages of multimorbid patients per physician discipline. No adjustment for age or SES was performed because of data restrictions. Although studies suggest that the number of multimorbid patients increases with age^19^^,^^21^^,^^22^ and correlates with low SES,^24^^,^^30^ they are not proven to be the main driver for healthcare utilization. Rather, it seems that the amount of healthcare utilization depends on the type and quantity of co-existing chronic diseases and other social determinants of health besides SES and age.^16^^,^^31^ Moreover, recent findings suggest that multimorbidity partially explains the need for healthcare independent of age and SES.^15^ When visually comparing clusters of our physician disciplines to the deprivation index of Kroll et al. (2017) of the year 2012, it appears that high-rate clusters correlate with a high levels of deprivation. Similarly, low-rate clusters in the south of Germany seem to correlate with low levels of deprivation. A direct comparison of our data with the data provided by Kroll et al.^32^ was not feasible as the regions used for their analysis are not compatible to the regions used for this article. Thus, additional analyses will be necessary to assess how age-/SES-specific multimorbidity rates influence the need for healthcare in Germany.

We compare the results of the spatial Bernoulli statistics between physician disciplines at area and cluster levels. In this context, it is important to note that area-level comparison bears the risk of introducing bias, especially in areas of large clusters, because all clusters are calculated by comparing the added risk within certain areas with the risk outside these areas. As the main aim of our article is to compare variations in multimorbidity levels between physician disciplines for planning purposes and not to compare individual risk ratios at area level, we also consider displaying a comparison of high-rate areas appropriate.

One limitation when defining multimorbidity through claims data is that they are recorded for billing purposes in outpatient care and thus might not reflect the true underlying multimorbidity as the data are dependent on supply. Additionally, uninsured individuals are not represented in our dataset. Consequently, they inherit a source bias that is not accounted for in this study because of lack of appropriate data. If additional epidemiological data are available, future studies may compare the results derived from secondary data with epidemiological data to externally validate the data source.

### Implications for policy and research

Previous research has shown that particularly multimorbid patients face the consequences of the fragmentation of ambulatory care to a higher degree, suffer greater risks of adverse effects resulting from polypharmacy and report a reduced overall quality of life,^7^^,^^33^ which is aggravated by the increasing prevalence of multiple chronic conditions. Likewise, physicians reported challenges in treating multimorbid patients, such as lacking evidence of practice, again fragmented healthcare systems and clinical uncertainty linked to multimorbidity.^34^ Thus, the outcomes of our article can be used by policymakers to reform current workforce planning by strengthening care specifically in areas with high rates of multimorbid individuals and, thus, improving the quality of office-based care for both patients and healthcare providers.

## Supplementary Material

ckad039_Supplementary_Data

## Data Availability

The multimorbidity data underlying this article cannot be shared publicly as they were provided by the German National Association of Statutory Health Insurance Physicians and underly their permission. To our knowledge, this study is the first to use nationwide claims data without age restriction to compare physician-specific multimorbidity levels at a regional level. Although multimorbidity levels vary greatly between physicians, high-rate areas were found to overlap frequently. Our results can be applied by policymakers to reform current workforce planning and thus improve the quality of ambulatory care for both patients and physicians. Future research regarding the influence of multimorbidity on patient visits and on consultation lengths differentiated by physician discipline can complement our findings and help to put our results into practice.
