# Supplementary material for: Regional variations in multimorbidity burden among office-based physicians in Germany
Source: Eur J Public Health. 2023 Mar 15;33(3):389–95. doi: 10.1093/eurpub/ckad039 (PMC10234650; doi:10.1093/eurpub/ckad039)
Supplement: ckad039_Supplementary_Data [file ckad039_supplementary_data.zip › ckad039_Supplementary_Data/ejph-2022-10-om-0501-File006.pdf]

Supplementary file 1: Multimorbidity categories recommended by Barnett et al. (2012)[1] including respective ICD-10-GM codes

| Disease categories | ICD-10-GM codes | | |
|---|---|---|---|
| | 1-digit code | 3-digit code | 4/5-digit code |
| **Hypertension** | | I10 - I11 | |
| | | O11 | |
| **Depression** | | F33 | F41.2 |
| | | F32 | F20.4 |
| | | F34 | |
| **Painful condition** | | M54 | F62.80 |
| | | N23 | F45.40 |
| | | R51, R52 | H57.1 |
| | | R10 | K14.6 |
| | | | K08.88 |
| | | | M25.5 |
| | | | M79.6 |
| | | | N64.4 |
| | | | H92.0 |
| | | | R10.2 |
| | | | R07.0, R07.1 |
| **Asthma (currently treated)** | | J45, J46 | |
| **Coronary heart disease** | | I20 - I25 | |
| **Treated dyspepsia** | | K29 - K31 | F45.31 |
| | | | R10.1 |
| **Diabetes** | | E10 - E14 | |
| **Thyroid disorders** | | E00 - E07 | |
| **Rheumatoid arthritis, other inflammatory polyarthropathies & systematic connective tissue disorders** | | M05 - M14 | |
| | | M20 - M25 | |
| **Hearing loss** | | H90 | H83.3 |
| | | H93 | H91.0, H91.2, H91.3, H91.9 |
| **Chronic obstructive pulmonary disease** | | J44 | |
| **Anxiety & other neurotic, stress related & somatoform disorders** | | F40 - F48 | |
| **Irritable bowel syndrome** | | K58 | |
| **New diagnosis of cancer in last five years** | C, D (48) | | |
| **Alcohol problems** | | F10 | |
| **Other psychoactive substance misuse** | | F11 - F19 | |
| **Treated constipation** | | | K59.0 |
| **Stroke & transient ischaemic attack** | | I60 - I62 | |
| | | I63 | |
| | | I65 - I67 | |
| | | G45, G46 | |
| **Chronic kidney disease** | | N18 | |
| **Diverticular disease of intestine** | | K57 | |
| **Atrial fibrillation** | | I47 - I49 | |
| **Peripheral vascular disease** | | I70 - I89 | |
| **Heart failure** | | I50 | |
| **Prostate disorders** | | N40 - N42 | |
| **Glaucoma** | | H40 | |
| **Epilepsy (currently treated)** | | G40, G41 | |
| **Dementia** | | F00 - F03 | |
| **Schizophrenia (and related non-organic psychosis) or bipolar disorder** | | F20, F21, F25 | F23.3 |
| | | | F20.4 |
| **Psoriasis or eczema** | | L40 | |
| | | L20, L21 | |
| **Inflammatory bowel disease** | | K50 - K52 | |
| **Migraine** | | G43, G44 | |
| **Blindness & low vision** | | H53, H54 | |
| **Chronic sinusitis** | | J32 | |
| **Learning disability** | | F80, F81, F83, F84 | |
| **Anorexia or bulimia** | | F50 | |
| **Bronchiectasis** | | J47 | Q33.4 |
| | | A15, A16 | |
| **Parkinson's disease** | | G20, G22 | |
| | | G21 | |
| **Multiple sclerosis** | | G35 | R63.0 |
| **Viral Hepatitis** | | B15 - B19 | |
| **Chronic liver disease** | | K70-77 | |

1. Barnett K, Mercer SW, Norbury M, et al. Epidemiology of multimorbidity and implications for health care, research, and medical education: a cross-sectional study. The Lancet 2012; 380: 37–43.
