# Supplementary material for: Regional variations in multimorbidity burden among office-based physicians in Germany
Source: Eur J Public Health. 2023 Mar 15;33(3):389–95. doi: 10.1093/eurpub/ckad039 (PMC10234650; doi:10.1093/eurpub/ckad039)
Supplement: ckad039_Supplementary_Data [file ckad039_supplementary_data.zip › ckad039_Supplementary_Data/ejph-2022-10-om-0501-File007.pdf]

Supplementary file 2

Results for Bernoulli purely spatial analysis scanning for clusters with high and low rates

General information:

Data Checking

Temporal Data Check: Check to ensure that all cases and controls are within the specified temporal study period.
Geographical Data Check: Check to ensure that all observations (cases, controls and populations) are within the specified geographical area.

Spatial Neighbours

Use Non-Euclidian Neighbours file: No
Use Meta Locations File: No
Multiple Coordinates Type: Allow only one set of coordinates per location ID.

Spatial Window

Maximum Spatial Cluster Size 50 percent of population at risk
Window Shape: Circular
Isotonic Scan: No

Inference

P-Value Reporting: Standard Monte Carlo
Report Gumbel Based P-Values: No
Number of Replications: 999
Adjusting for More Likely Clusters: No

Spatial Output

Report Hierarchical Clusters: Yes
Criteria for Reporting Secondary Clusters: No Geographical Overlap
Report Gini Optimized Cluster Collection: No
Restrict Reporting to Smaller Clusters: Yes
Reported Clusters: Only clusters smaller than 10 percent of population at risk reported.

1. **GPs**

SUMMARY OF DATA

Study period: 2015/1/1 to 2015/12/31
Number of locations: 959
Total population: 54,799,570
Total number of cases: 17,239,488
Percent cases in area: 31.5

CLUSTERS DETECTED

1. Location IDs included.: 3400200, 3400196, 3400407, 3400207, 3400201, 3400206, 3400194, 1204120, 3400164, 3400202, 3400409, 1204070, 3400321, 3400198, 3400204, 1503070, 3400208, 3400165, 3400203, 3400193, 1204080, 1204110, 3400166, 1603000, 3400167,

Supplementary file 2

3400209, 3400190, 1503010, 3400178, 1503020, 3400172, 3400189, 3400188, 1603100, 1205080, 3400192, 3400171, 1504020, 1504030, 3400168, 1204100, 3400177, 3500161, 3500159, 1503060, 3400173, 3400191, 1204040, 3400176, 3400199, 3600522, 3400169, 3400197, 3400187, 1503040, 1504040, 3600526, 1205070, 1204020, 1602600, 3400170, 1204090, 3400408, 1205110, 1602900, 1504010, 1204050, 1205050, 1602700, 3400186, 1503030, 3600527, 1503050, 3400174, 1603500, 1602500, 1204010, 1602000, 3600524, 1205120, 1504060, 1502020, 1205130, 3400181, 3400179, 1204060, 3400185, 1205040, 1205100, 1603400, 3600528, 1204030, 1204130, 1502050, 1203040, 3400182, 3400180, 1505050, 1505060, 1600200, 1603200, 1205030, 3400184, 1505040, 1601900, 3400183, 3500162

Coordinates / radius..: (51.319400 N, 13.053642 E) / 131.52 km
Population............: 5428136
Number of cases.......: 2117537
Expected cases........: 1709101.26
Observed / expected...: 1.24
Relative risk.........: 1.27
Percent cases in area.: 39.0
Log likelihood ratio..: 76489.133840
Monte Carlo rank......: 1/1000
P-value...............: 0.001

2. Location IDs included.: 811050, 811020, 915030, 811040, 915020, 811030, 2900144, 811060, 811010, 812080, 915040, 915010, 2900145, 810060, 812040, 915050, 812020, 801140, 810030, 812030, 803040, 812010, 2900143, 812070, 915060, 801130, 2900352, 801100, 2900391, 810020, 916030, 916010, 801120, 812090, 2900123, 812060, 916020, 916050, 810080, 803030, 812050, 801090, 2900343, 916060, 810050, 810010, 803020, 801070, 2900142, 916070, 2900371, 809060, 2900309, 2900373, 2900122, 801060, 810040, 809090, 810070, 801080, 801110, 909050, 2900303, 803010, 808040, 916040, 917010, 801150, 809080, 801030, 914050, 909040, 2900302, 2900310, 916080, 806070, 801040, 809070, 802090, 801050, 808020, 917020, 914080, 2900124, 806050, 910030, 806040, 801010, 801020, 914030, 2900152, 910040, 917080, 802050, 808050, 2900132, 808010, 802080, 917040, 806060

Coordinates / radius..: (48.212343 N, 9.925358 E) / 115.75 km
Population............: 5329548
Number of cases.......: 1404320
Expected cases........: 1678059.87
Observed / expected...: 0.84
Relative risk.........: 0.82
Percent cases in area.: 26.3
Log likelihood ratio..: 37164.729538
Monte Carlo rank......: 1/1000
P-value...............: 0.001

3. Location IDs included.: 3300503, 3300501, 3300511, 3300502, 3300505, 3300520, 3300509, 3300510, 3300506, 3300514, 3300516, 1302050, 1303070, 3300513, 1303080, 3300517, 3300515, 3300518, 1302040, 3300507, 1202010, 1202020, 1201080, 1301060, 1202030, 3300508, 1201010, 1201040, 3300155, 1202040, 3300519, 1201050, 1201060, 1202050, 1203060, 1201030, 3300512, 1201070, 3300504

Coordinates / radius..: (54.088914 N, 13.414215 E) / 157.82 km
Population............: 1665881
Number of cases.......: 656200
Expected cases........: 524518.79
Observed / expected...: 1.25
Relative risk.........: 1.26
Percent cases in area.: 39.4
Log likelihood ratio..: 23961.215522
Monte Carlo rank......: 1/1000
P-value...............: 0.001

Supplementary file 2

4. Location IDs included.: 2400001, 339010, 2300429, 2300434, 2300087, 2300107, 2300112, 333020, 314020, 319010, 2300108, 2300100, 2300103, 2400000, 2300105, 321010, 2300099, 319020, 329010, 2300086, 2300115, 2300090, 104030, 2300085, 321020, 2300111, 330020, 2300101, 104020, 331010, 319030, 104040, 2300442, 323020, 2100083, 104010, 330010, 2300102, 338010, 315010, 105070, 331020, 2300096, 2200000, 307020, 325010, 332010, 320010, 2300437, 307030, 103020, 103040, 320030, 338020, 2300113, 2100080, 337010, 2300441, 2100084, 320020, 105050, 332020, 101120, 504010, 306010, 101110, 337020, 504020, 2300004, 103010, 2300114, 2300432, 2100082, 306030, 101090

Coordinates / radius..: (53.542867 N, 8.576513 E) / 134.61 km
Population............: 5401922
Number of cases.......: 1531117
Expected cases........: 1700847.53
Observed / expected...: 0.90
Relative risk.........: 0.89
Percent cases in area.: 28.3
Log likelihood ratio..: 13948.338567
Monte Carlo rank......: 1/1000
P-value...............: 0.001

5. Location IDs included.: 1600900, 601200, 1601100, 601210, 1600800, 1601000, 2900372, 601190, 601180, 601220, 601170, 2900346, 3600523, 1601200, 601130, 903020, 601150, 602080, 2900345, 1601800, 1600500, 1601300, 2600270, 601120, 903030, 1600400, 603060, 2600272, 1601500, 1600600, 602070, 601050, 3600521, 903040, 2600271, 2900148, 601100, 2600274, 603090, 602060, 2900127, 2900304, 2900149, 904020, 602120, 2600273, 1602300, 1600700, 1600300, 603070, 2300095, 1602200, 904010, 601090

Coordinates / radius..: (50.745283 N, 10.128848 E) / 84.64 km
Population............: 2197553
Number of cases.......: 773317
Expected cases........: 691920.87
Observed / expected...: 1.12
Relative risk.........: 1.12
Percent cases in area.: 35.2
Log likelihood ratio..: 7143.345783
Monte Carlo rank......: 1/1000
P-value...............: 0.001

6. Location IDs included.: 603130, 603110, 603160, 603170, 603140, 603100, 603120, 703030, 603190, 603260, 703020, 603150, 603200, 603180, 703010, 603250, 603020, 701110, 2700116, 703050, 602150, 603270

Coordinates / radius..: (50.079149 N, 8.260376 E) / 38.74 km
Population............: 1974684
Number of cases.......: 550963
Expected cases........: 621748.41
Observed / expected...: 0.89
Relative risk.........: 0.88
Percent cases in area.: 27.9
Log likelihood ratio..: 6231.632185
Monte Carlo rank......: 1/1000
P-value...............: 0.001

Supplementary file 2

7. Location IDs included.: 503060, 503050, 503120, 503040, 503110, 503130, 503070, 503140, 503170, 503010, 503080, 503160, 503090, 503150, 503100, 503230, 2300438, 503180, 503240, 332030, 503020, 503270

Coordinates / radius..: (52.093125 N, 7.058568 E) / 49.60 km
Population............: 1137345
Number of cases.......: 319570
Expected cases........: 358104.10
Observed / expected...: 0.89
Relative risk.........: 0.89
Percent cases in area.: 28.1
Log likelihood ratio..: 3153.444098
Monte Carlo rank......: 1/1000
P-value...............: 0.001

8. Location IDs included.: 911050, 911060, 2900350, 912010, 2900386, 2900401, 2900406, 2900377, 912090, 2900383, 2900351, 2900400, 2900392, 2900397, 2900394, 2900375, 911030, 912100, 2900362, 906070, 2900379, 2900384, 2900357, 2900141, 2900398, 2900385, 2900344, 2900341, 912050, 912060, 2900396, 906020, 913030, 2900367, 2900128, 2900353, 911070, 911090, 2900380, 906010, 2900359, 906040, 911080, 2900368, 2900356, 2900361, 2900146, 2900129, 2900370, 912080, 913080, 2900358, 913010, 911010, 2900402, 907060, 2900393, 913070, 905040, 905080, 2900136, 914010, 2900399, 2900138, 918010, 907050, 2900342, 2900348, 2900135, 2900130, 918020, 2900137

Coordinates / radius..: (49.285830 N, 12.880936 E) / 123.30 km
Population............: 2243965
Number of cases.......: 659238
Expected cases........: 706534.14
Observed / expected...: 0.93
Relative risk.........: 0.93
Percent cases in area.: 29.4
Log likelihood ratio..: 2438.324057
Monte Carlo rank......: 1/1000
P-value...............: 0.001

9. Location IDs included.: 501430, 501400, 505360, 501420, 501440, 505340, 501390, 501160, 501410, 505350, 501380, 501170, 501370, 505390, 505320, 505330, 501460, 505370, 501450, 501360

Coordinates / radius..: (51.337479 N, 7.088029 E) / 21.23 km
Population............: 1573763
Number of cases.......: 533963
Expected cases........: 495514.54
Observed / expected...: 1.08
Relative risk.........: 1.08
Percent cases in area.: 33.9
Log likelihood ratio..: 2212.714316
Monte Carlo rank......: 1/1000
P-value...............: 0.001

Supplementary file 2

10. Location IDs included.: 705030, 705020, 705040, 1000080, 705050, 705060
   Coordinates / radius..: (49.413043 N, 7.526107 E) / 20.80 km
   Population............: 340205
   Number of cases.......: 125115
   Expected cases........: 107116.84
   Observed / expected...: 1.17
   Relative risk.........: 1.17
   Percent cases in area.: 36.8
   Log likelihood ratio..: 2161.111783
   Monte Carlo rank......: 1/1000
   P-value...............: 0.001

11. Location IDs included.: 505160, 505170, 505130, 505190, 505200, 505150, 505140, 505180, 701010, 602020, 505120, 505230, 505210, 502300, 602010, 502330, 505090, 505240, 602090, 601070, 505070
   Coordinates / radius..: (50.984411 N, 8.061247 E) / 38.92 km
   Population............: 754396
   Number of cases.......: 212675
   Expected cases........: 237528.90
   Observed / expected...: 0.90
   Relative risk.........: 0.89
   Percent cases in area.: 28.2
   Log likelihood ratio..: 1962.870483
   Monte Carlo rank......: 1/1000
   P-value...............: 0.001

12. Location IDs included.: 504220, 504210, 505020, 504230, 504200
   Coordinates / radius..: (51.765423 N, 8.540728 E) / 20.21 km
   Population............: 266785
   Number of cases.......: 70591
   Expected cases........: 83999.84
   Observed / expected...: 0.84
   Relative risk.........: 0.84
   Percent cases in area.: 26.5
   Log likelihood ratio..: 1620.065221
   Monte Carlo rank......: 1/1000
   P-value...............: 0.001

13. Location IDs included.: 306050
   Coordinates / radius..: (52.465977 N, 9.704680 E) / 0 km
   Population............: 37589
   Number of cases.......: 15180
   Expected cases........: 11835.26
   Observed / expected...: 1.28
   Relative risk.........: 1.28
   Percent cases in area.: 40.4
   Log likelihood ratio..: 661.230403
   Monte Carlo rank......: 1/1000
   P-value...............: 0.001

Supplementary file 2

14. Location IDs included.: 902050, 2900389, 903070, 2900403, 2900313, 2900404
   Coordinates / radius..: (49.753176 N, 10.256027 E) / 22.89 km
   Population............: 227996
   Number of cases.......: 64713
   Expected cases........: 71786.75
   Observed / expected...: 0.90
   Relative risk.........: 0.90
   Percent cases in area.: 28.4
   Log likelihood ratio..: 520.489293
   Monte Carlo rank......: 1/1000
   P-value...............: 0.001

15. Location IDs included.: 502120, 502080, 502110, 502220, 502070, 502100, 502230, 502130, 502060, 502180, 502050, 502030, 502140, 502240, 502020, 502170, 502040, 502160, 502010, 502190, 501320, 502380, 502260, 501260, 502150, 502390, 501250, 502200, 502400, 501270, 502250, 501330, 502420, 501230, 501290, 501280, 502410, 501240
   Coordinates / radius..: (50.828460 N, 6.269418 E) / 51.32 km
   Population............: 1742905
   Number of cases.......: 568218
   Expected cases........: 548770.54
   Observed / expected...: 1.04
   Relative risk.........: 1.04
   Percent cases in area.: 32.6
   Log likelihood ratio..: 516.342800
   Monte Carlo rank......: 1/1000
   P-value...............: 0.001

16. Location IDs included.: 502430
   Coordinates / radius..: (50.688724 N, 7.095696 E) / 0 km
   Population............: 199568
   Number of cases.......: 56252
   Expected cases........: 62835.92
   Observed / expected...: 0.90
   Relative risk.........: 0.89
   Percent cases in area.: 28.2
   Log likelihood ratio..: 515.520854
   Monte Carlo rank......: 1/1000
   P-value...............: 0.001

17. Location IDs included.: 505430, 505420, 505460, 505450, 505400, 505440, 503290
   Coordinates / radius..: (51.622922 N, 7.629610 E) / 15.89 km
   Population............: 374967
   Number of cases.......: 126716
   Expected cases........: 118062.00
   Observed / expected...: 1.07
   Relative risk.........: 1.07
   Percent cases in area.: 33.8
   Log likelihood ratio..: 460.306138
   Monte Carlo rank......: 1/1000
   P-value...............: 0.001

Supplementary file 2

18. Location IDs included.: 907010, 907020, 2900364, 904060, 907030, 2900378, 2900347, 2900349, 907040
  Coordinates / radius..: (49.666190 N, 10.914525 E) / 28.93 km
  Population............: 927128
  Number of cases.......: 304870
  Expected cases........: 291915.24
  Observed / expected...: 1.04
  Relative risk.........: 1.05
  Percent cases in area.: 32.9
  Log likelihood ratio..: 423.526710
  Monte Carlo rank......: 1/1000
  P-value...............: 0.001

19. Location IDs included.: 701050
  Coordinates / radius..: (50.471370 N, 6.999173 E) / 0 km
  Population............: 63772
  Number of cases.......: 22953
  Expected cases........: 20079.23
  Observed / expected...: 1.14
  Relative risk.........: 1.14
  Percent cases in area.: 36.0
  Log likelihood ratio..: 293.484437
  Monte Carlo rank......: 1/1000
  P-value...............: 0.001

20. Location IDs included.: 2700283, 2700281, 702080, 2700282, 702050, 1000010, 702060, 702040, 1000020, 703080
  Coordinates / radius..: (49.786803 N, 6.746398 E) / 39.02 km
  Population............: 377791
  Number of cases.......: 125826
  Expected cases........: 118951.16
  Observed / expected...: 1.06
  Relative risk.........: 1.06
  Percent cases in area.: 33.3
  Log likelihood ratio..: 289.077760
  Monte Carlo rank......: 1/1000
  P-value...............: 0.001

2. Ophthalmologists

SUMMARY OF DATA

Study period: 2015/1/1 to 2015/12/31
Number of locations: 385
Total population: 16,195,148
Total number of cases: 7,145,558
Percent cases in area: 44.1

Supplementary file 2

CLUSTERS DETECTED

1. Location IDs included.: 150840, 160740, 150880, 160520, 160530, 150020, 340061, 340021, 160710, 160770, 160760, 160680, 150870, 340033, 340035, 340024, 160510, 340039, 160750, 160650, 160730, 350017, 340027, 160700, 150890, 340019, 340083, 340028, 350018, 340081, 340036, 160670, 160620, 340038, 160640, 150910, 160720, 94750, 94760, 150850, 340023, 340026, 340037, 150030, 340022, 160690, 340082, 160610, 160660

   Coordinates / radius..: (51.147303 N, 11.883881 E) / 117.24 km
   Population............: 1556108
   Number of cases.......: 883094
   Expected cases........: 686579.75
   Observed / expected...: 1.29
   Relative risk.........: 1.33
   Percent cases in area.: 56.8
   Log likelihood ratio..: 55229.122868
   Monte Carlo rank......: 1/1000
   P-value...............: 0.001

2. Location IDs included.: 130620, 120730, 130590, 130550, 130520, 120600, 120650, 130560, 130610, 120640, 130570, 120680, 130530, 310016, 130510, 130030, 120630, 130600, 120540, 120670, 120700, 120610, 120690, 120720, 150900, 130580, 130540, 150860, 120520, 120710

   Coordinates / radius..: (53.574148 N, 14.069925 E) / 203.26 km
   Population............: 1604093
   Number of cases.......: 845977
   Expected cases........: 707751.50
   Observed / expected...: 1.20
   Relative risk.........: 1.22
   Percent cases in area.: 52.7
   Log likelihood ratio..: 26608.229601
   Monte Carlo rank......: 1/1000
   P-value..............: 0.001

3. Location IDs included.: 84260, 84360, 84250, 84210, 97750, 84370, 84150, 97780, 84350, 97740, 97760, 81170, 84170, 81160, 81350, 97800, 84160, 83350, 83270, 97770, 97720, 97730, 81110, 97610, 91810, 81150, 81360, 81190, 83250, 97710, 91900, 83260, 97790, 81180, 82370, 82350, 91790, 91880, 81270

   Coordinates / radius..: (48.107673 N, 9.774403 E) / 115.62 km
   Population............: 1532075
   Number of cases.......: 546811
   Expected cases........: 675976.01
   Observed / expected...: 0.81
   Relative risk.........: 0.79
   Percent cases in area.: 35.7
   Log likelihood ratio..: 24803.581092
   Monte Carlo rank......: 1/1000
   P-value..............: 0.001

Supplementary file 2

4. Location IDs included.: 92760, 92710, 92720, 93720, 92780, 92750, 92790, 92770, 93750, 93620, 93760, 92740, 92730, 91710, 91830, 93740, 93710, 93730, 91770, 93770, 91780, 91860, 91760, 91610, 91890, 95740, 91750, 94790, 91850, 91870, 95760, 94720, 91720, 91740, 91620, 91840
  Coordinates / radius..: (49.022814 N, 13.099907 E) / 149.87 km
  Population............: 1180312
  Number of cases.......: 455320
  Expected cases........: 520772.54
  Observed / expected...: 0.87
  Relative risk.........: 0.87
  Percent cases in area.: 38.6
  Log likelihood ratio..: 8020.783563
  Monte Carlo rank......: 1/1000
  P-value...............: 0.001

5. Location IDs included.: 10610, 10560, 10510, 33590, 10600, 10580, 20000, 33520, 10620, 10020, 10570, 33530, 40120, 33570
  Coordinates / radius..: (53.924334 N, 9.514041 E) / 76.12 km
  Population............: 897025
  Number of cases.......: 340739
  Expected cases........: 395781.78
  Observed / expected...: 0.86
  Relative risk.........: 0.85
  Percent cases in area.: 38.0
  Log likelihood ratio..: 7337.090564
  Monte Carlo rank......: 1/1000
  P-value...............: 0.001

6. Location IDs included.: 55660, 55150, 34040, 34590, 55580, 55700, 34560, 55540, 34540, 57540, 59150, 34600, 55620
  Coordinates / radius..: (52.211514 N, 7.579225 E) / 66.45 km
  Population............: 761920
  Number of cases.......: 286515
  Expected cases........: 336171.30
  Observed / expected...: 0.85
  Relative risk.........: 0.85
  Percent cases in area.: 37.6
  Log likelihood ratio..: 6975.807202
  Monte Carlo rank......: 1/1000
  P-value...............: 0.001

7. Location IDs included.: 100440, 100420, 100410, 100430, 100460
  Coordinates / radius..: (49.355298 N, 6.775427 E) / 29.75 km
  Population............: 161830
  Number of cases.......: 54027
  Expected cases........: 71401.99
  Observed / expected...: 0.76
  Relative risk.........: 0.75
  Percent cases in area.: 33.4
  Log likelihood ratio..: 3919.720863
  Monte Carlo rank......: 1/1000
  P-value...............: 0.001

Supplementary file 2

8. Location IDs included.: 64370, 96760, 64310, 64320, 82250, 64110, 82210, 82260, 82220, 96710, 64380, 64330, 73140, 64130, 73380, 81280, 64120, 81250, 73310, 81210, 96770, 64360, 73150, 81260, 64350, 73320, 64140, 96790, 96630, 73390, 82150, 64340, 64400
   Coordinates / radius..: (49.671448 N, 8.979515 E) / 76.11 km
   Population............: 1581231
   Number of cases.......: 656609
   Expected cases........: 697664.42
   Observed / expected...: 0.94
   Relative risk.........: 0.94
   Percent cases in area.: 41.5
   Log likelihood ratio..: 2405.610518
   Monte Carlo rank......: 1/1000
   P-value...............: 0.001

9. Location IDs included.: 59700, 59660, 65320, 71320, 65340, 59580, 53740
   Coordinates / radius..: (50.937659 N, 8.194734 E) / 48.15 km
   Population............: 282308
   Number of cases.......: 113798
   Expected cases........: 124558.81
   Observed / expected...: 0.91
   Relative risk.........: 0.91
   Percent cases in area.: 40.3
   Log likelihood ratio..: 852.522449
   Monte Carlo rank......: 1/1000
   P-value...............: 0.001

10. Location IDs included.: 51110
   Coordinates / radius..: (51.235418 N, 6.810261 E) / 0 km
   Population............: 105254
   Number of cases.......: 40695
   Expected cases........: 46439.75
   Observed / expected...: 0.88
   Relative risk.........: 0.88
   Percent cases in area.: 38.7
   Log likelihood ratio..: 646.961954
   Monte Carlo rank......: 1/1000
   P-value...............: 0.001

11. Location IDs included.: 32411, 32412, 31570, 32540, 32570, 32520, 33510, 31020, 32560, 31010, 32550, 31510, 33580, 31580, 31030, 57700, 31530, 31550
   Coordinates / radius..: (52.379486 N, 9.769642 E) / 70.98 km
   Population............: 790463
   Number of cases.......: 362014
   Expected cases........: 348764.92
   Observed / expected...: 1.04
   Relative risk.........: 1.04
   Percent cases in area.: 45.8
   Log likelihood ratio..: 472.367233
   Monte Carlo rank......: 1/1000
   P-value...............: 0.001

Supplementary file 2

12. Location IDs included.: 59110
   Coordinates / radius..: (51.469928 N, 7.224923 E) / 0 km
   Population............: 82982
   Number of cases.......: 40225
   Expected cases........: 36612.99
   Observed / expected...: 1.10
   Relative risk.........: 1.10
   Percent cases in area.: 48.5
   Log likelihood ratio..: 318.710764
   Monte Carlo rank......: 1/1000
   P-value...............: 0.001

13. Location IDs included.: 94710, 95720, 96740, 94740, 94780
   Coordinates / radius..: (49.894622 N, 10.893297 E) / 28.70 km
   Population............: 117407
   Number of cases.......: 47664
   Expected cases........: 51801.85
   Observed / expected...: 0.92
   Relative risk.........: 0.92
   Percent cases in area.: 40.6
   Log likelihood ratio..: 299.837851
   Monte Carlo rank......: 1/1000
   P-value...............: 0.001

14. Location IDs included.: 51140, 51120, 51660
   Coordinates / radius..: (51.345236 N, 6.579665 E) / 18.94 km
   Population............: 183259
   Number of cases.......: 85623
   Expected cases........: 80856.80
   Observed / expected...: 1.06
   Relative risk.........: 1.06
   Percent cases in area.: 46.7
   Log likelihood ratio..: 253.351019
   Monte Carlo rank......: 1/1000
   P-value...............: 0.001

3. Orthopaedic specialist

SUMMARY OF DATA

Study period.......................: 2015/1/1 to 2015/12/31
Number of locations................: 385
Total population...................: 11,659,090
Total number of cases..............: 4,722,933
Percent cases in area..............: 40.5

Supplementary file 2

CLUSTERS DETECTED

1. Location IDs included.: 340032, 340020, 340037, 340031, 340022, 340026, 340082, 340081, 340027, 340019, 340029, 340023, 340042, 340028, 340024, 340043, 340035, 340036, 340038, 120620, 120660, 340039, 160770, 340061, 340041, 340021, 340033, 160520, 120520, 340083, 120710, 160760, 150910, 150840, 120610, 120720, 150020, 160740, 150880, 160750, 94790, 160530, 350017, 350018, 94750, 93770, 120670, 160710, 120690, 160730, 94760, 93740, 150870, 150890, 120540, 94770

Coordinates / radius..: (50.890262 N, 13.650447 E) / 176.50 km
Population............: 1138830
Number of cases.......: 600906
Expected cases........: 461324.02
Observed / expected...: 1.30
Relative risk.........: 1.35
Percent cases in area.: 52.8
Log likelihood ratio..: 38598.865331
Monte Carlo rank......: 1/1000
P-value...............: 0.001

2. Location IDs included.: 84150, 81160, 84160, 84250, 84170, 81170, 84370, 84210, 81110, 81150, 84260, 81190, 97750, 83270, 82350, 81180, 83250, 81350, 82370, 82310, 84360, 82360, 81360, 84350, 97740, 83350, 83260, 81210, 97780, 81250, 97730, 81270

Coordinates / radius..: (48.406387 N, 9.365822 E) / 91.13 km
Population............: 1062119
Number of cases.......: 344208
Expected cases........: 430249.47
Observed / expected...: 0.80
Relative risk.........: 0.78
Percent cases in area.: 32.4
Log likelihood ratio..: 16297.499237
Monte Carlo rank......: 1/1000
P-value...............: 0.001

3. Location IDs included.: 130520, 130550, 130590, 130560, 130530, 130570, 130510, 130030, 130620, 130610, 130600, 120730, 120680, 120650, 120700, 120600, 130580, 130540

Coordinates / radius..: (53.797036 N, 13.041971 E) / 130.09 km
Population............: 322937
Number of cases.......: 174974
Expected cases........: 130817.24
Observed / expected...: 1.34
Relative risk.........: 1.35
Percent cases in area.: 54.2
Log likelihood ratio..: 12604.469461
Monte Carlo rank......: 1/1000
P-value...............: 0.001

Supplementary file 2

4. Location IDs included.: 10610, 10560, 10510, 33590, 10600, 10580, 20000, 33520, 10620, 10020, 10570, 33530, 40120, 33570
  Coordinates / radius..: (53.924334 N, 9.514041 E) / 76.12 km
  Population............: 630204
  Number of cases.......: 205382
  Expected cases........: 255286.78
  Observed / expected...: 0.80
  Relative risk.........: 0.80
  Percent cases in area.: 32.6
  Log likelihood ratio..: 8881.998704
  Monte Carlo rank......: 1/1000
  P-value...............: 0.001

5. Location IDs included.: 91840, 91620, 91750, 91880, 91790, 91740, 91770, 91820, 91730, 91780, 91870, 91810, 91900, 91830, 91860, 97710
  Coordinates / radius..: (48.077661 N, 11.646389 E) / 58.65 km
  Population............: 569294
  Number of cases.......: 189205
  Expected cases........: 230612.99
  Observed / expected...: 0.82
  Relative risk.........: 0.81
  Percent cases in area.: 33.2
  Log likelihood ratio..: 6716.969712
  Monte Carlo rank......: 1/1000
  P-value...............: 0.001

6. Location IDs included.: 66320, 66340, 160630, 66360, 66310, 65350, 66110, 160660, 66330, 160610, 160640, 160670, 31520, 66350, 96730, 65340, 65310, 96720, 64350, 64400, 160690, 160700, 160510, 31550, 160620, 57620, 31560, 160650, 65320, 160680
  Coordinates / radius..: (50.906127 N, 9.752800 E) / 101.88 km
  Population............: 623733
  Number of cases.......: 285133
  Expected cases........: 252665.47
  Observed / expected...: 1.13
  Relative risk.........: 1.14
  Percent cases in area.: 45.7
  Log likelihood ratio..: 3664.122625
  Monte Carlo rank......: 1/1000
  P-value...............: 0.001

7. Location IDs included.: 100440, 100420, 100410, 100430, 100460
  Coordinates / radius..: (49.355298 N, 6.775427 E) / 29.75 km
  Population............: 138825
  Number of cases.......: 41446
  Expected cases........: 56236.06
  Observed / expected...: 0.74
  Relative risk.........: 0.73
  Percent cases in area.: 29.9
  Log likelihood ratio..: 3433.147290
  Monte Carlo rank......: 1/1000
  P-value...............: 0.001

Supplementary file 2

8. Location IDs included.: 34040, 34590, 55660, 57580, 57540, 34600, 57110, 55700, 55150, 57700, 55580, 34540, 32510, 59150, 57660, 34530, 34560, 32570, 59740, 32560, 34580, 57740, 59780, 55540, 55620
  Coordinates / radius..: (52.277683 N, 8.047039 E) / 90.64 km
  Population............: 1113903
  Number of cases.......: 416396
  Expected cases........: 451226.44
  Observed / expected...: 0.92
  Relative risk.........: 0.92
  Percent cases in area.: 37.4
  Log likelihood ratio..: 2518.484320
  Monte Carlo rank......: 1/1000
  P-value...............: 0.001

9. Location IDs included.: 64330, 64110, 73150, 64360, 64320
  Coordinates / radius..: (49.904380 N, 8.470254 E) / 23.90 km
  Population............: 139233
  Number of cases.......: 47073
  Expected cases........: 56401.33
  Observed / expected...: 0.83
  Relative risk.........: 0.83
  Percent cases in area.: 33.8
  Log likelihood ratio..: 1340.113758
  Monte Carlo rank......: 1/1000
  P-value...............: 0.001

10. Location IDs included.: 53341, 53342, 53580, 53700, 53660
  Coordinates / radius..: (50.759551 N, 6.109727 E) / 45.08 km
  Population............: 194072
  Number of cases.......: 87073
  Expected cases........: 78615.84
  Observed / expected...: 1.11
  Relative risk.........: 1.11
  Percent cases in area.: 44.9
  Log likelihood ratio..: 769.947620
  Monte Carlo rank......: 1/1000
  P-value...............: 0.001

11. Location IDs included.: 51110
  Coordinates / radius..: (51.235418 N, 6.810261 E) / 0 km
  Population............: 114573
  Number of cases.......: 41097
  Expected cases........: 46411.91
  Observed / expected...: 0.89
  Relative risk.........: 0.88
  Percent cases in area.: 35.9
  Log likelihood ratio..: 523.760529
  Monte Carlo rank......: 1/1000
  P-value...............: 0.001

Supplementary file 2

12. Location IDs included.: 59660, 59700, 59620, 53740, 59580, 71320
Coordinates / radius..: (51.086125 N, 7.976392 E) / 40.68 km
Population............: 226743
Number of cases.......: 86407
Expected cases........: 91850.40
Observed / expected...: 0.94
Relative risk.........: 0.94
Percent cases in area.: 38.1
Log likelihood ratio..: 278.341399
Monte Carlo rank......: 1/1000
P-value...............: 0.001

13. Location IDs included.: 59110, 59160, 55130, 51130, 59540
Coordinates / radius..: (51.469928 N, 7.224923 E) / 15.31 km
Population............: 292449
Number of cases.......: 123359
Expected cases........: 118466.98
Observed / expected...: 1.04
Relative risk.........: 1.04
Percent cases in area.: 42.2
Log likelihood ratio..: 173.443781
Monte Carlo rank......: 1/1000
P-value...............: 0.001

14. Location IDs included.: 32520
Coordinates / radius..: (52.095088 N, 9.389877 E) / 0 km
Population............: 18239
Number of cases.......: 8530
Expected cases........: 7388.36
Observed / expected...: 1.15
Relative risk.........: 1.15
Percent cases in area.: 46.8
Log likelihood ratio..: 146.495503
Monte Carlo rank......: 1/1000
P-value...............: 0.001

15. Location IDs included.: 51700, 51190
Coordinates / radius..: (51.626927 N, 6.618260 E) / 20.24 km
Population............: 85248
Number of cases.......: 32292
Expected cases........: 34532.77
Observed / expected...: 0.94
Relative risk.........: 0.93
Percent cases in area.: 37.9
Log likelihood ratio..: 124.013962
Monte Carlo rank......: 1/1000
P-value...............: 0.001

Supplementary file 2

16. Location IDs included.: 51120, 51170
Coordinates / radius..: (51.439558 N, 6.734696 E) / 10.36 km
Population............: 103466
Number of cases.......: 44183
Expected cases........: 41912.62
Observed / expected...: 1.05
Relative risk.........: 1.05
Percent cases in area.: 42.7
Log likelihood ratio..: 103.732396
Monte Carlo rank......: 1/1000
P-value...............: 0.001

17. Location IDs included.: 71380, 71110, 71370, 53820, 71430
Coordinates / radius..: (50.557667 N, 7.469048 E) / 28.00 km
Population............: 192062
Number of cases.......: 80337
Expected cases........: 77801.62
Observed / expected...: 1.03
Relative risk.........: 1.03
Percent cases in area.: 41.8
Log likelihood ratio..: 70.372784
Monte Carlo rank......: 1/1000
P-value...............: 0.001

18. Location IDs included.: 53140
Coordinates / radius..: (50.705774 N, 7.109870 E) / 0 km
Population............: 49345
Number of cases.......: 18739
Expected cases........: 19988.97
Observed / expected...: 0.94
Relative risk.........: 0.94
Percent cases in area.: 38.0
Log likelihood ratio..: 66.443825
Monte Carlo rank......: 1/1000
P-value...............: 0.001

4. Neurologists

SUMMARY OF DATA

Study period: 2015/1/1 to 2015/12/31
Number of locations: 385
Total population: 4,386,298
Total number of cases: 2,637,461
Percent cases in area: 60.1

Supplementary file 2

CLUSTERS DETECTED

1. Location IDs included.: 340082, 340022, 120620, 340020, 340081, 340036, 340029, 120660, 340032, 340037, 340035, 340027, 340031, 340042, 340019, 340026, 340033, 340061, 340021, 150910, 120520, 340024, 120710, 340028, 120610, 120720, 160770, 340041, 340023, 340043, 340039, 340038, 350018, 150020, 350017, 160520, 150840, 150880, 120670, 160760, 120690, 120540

Coordinates / radius..: (51.311954 N, 13.494769 E) / 127.88 km
Population............: 437064
Number of cases.......: 305521
Expected cases........: 262804.59
Observed / expected...: 1.16
Relative risk.........: 1.18
Percent cases in area.: 69.9
Log likelihood ratio..: 9983.240277
Monte Carlo rank......: 1/1000
P-value...............: 0.001

2. Location IDs included.: 84150, 81160, 84160, 84250, 84170, 81170, 84370, 84210, 81110, 81150, 84260, 81190, 97750, 83270, 82350, 81180, 83250, 81350, 82370, 82310, 84360, 82360, 81360, 84350, 97740, 83350, 83260, 81210, 97780, 81250, 97730, 81270

Coordinates / radius..: (48.406387 N, 9.365822 E) / 91.13 km
Population............: 392866
Number of cases.......: 198564
Expected cases........: 236228.54
Observed / expected...: 0.84
Relative risk.........: 0.83
Percent cases in area.: 50.5
Log likelihood ratio..: 8122.802459
Monte Carlo rank......: 1/1000
P-value..............: 0.001

3. Location IDs included.: 130590, 130520, 130620, 130610, 130550, 130570, 120730, 130560, 130530, 130030, 130510, 120650, 120600, 120680, 130600, 120700, 120640, 130580

Coordinates / radius..: (53.940993 N, 13.662774 E) / 157.99 km
Population............: 175232
Number of cases.......: 125088
Expected cases........: 105366.20
Observed / expected...: 1.19
Relative risk.........: 1.20
Percent cases in area.: 71.4
Log likelihood ratio..: 5022.797305
Monte Carlo rank......: 1/1000
P-value..............: 0.001

Supplementary file 2

4. Location IDs included.: 92750, 92720, 92710, 92770, 92760, 92790, 91710, 92780, 91830, 92740, 93720, 91890, 91720, 93620, 91770, 93750, 92730, 91870, 91780, 91750, 93760, 91860, 91840, 91620, 91610, 91820, 91740, 93730, 91760, 93710, 93740, 91850, 91790, 91880, 91730, 97710, 93770, 95740, 95760, 97610, 95770, 91810, 91900
   Coordinates / radius..: (48.559606 N, 13.368473 E) / 192.15 km
   Population............: 424221
   Number of cases.......: 236621
   Expected cases........: 255082.15
   Observed / expected...: 0.93
   Relative risk.........: 0.92
   Percent cases in area.: 55.8
   Log likelihood ratio..: 1837.135196
   Monte Carlo rank......: 1/1000
   P-value...............: 0.001

5. Location IDs included.: 160660, 96730, 160690, 160630, 160670, 160700, 66310, 94730, 160720, 96720, 66320, 160510, 160730, 160640, 96740, 96780, 66360, 94760, 94780, 160710, 160680, 65350, 160610
   Coordinates / radius..: (50.629134 N, 10.427062 E) / 84.72 km
   Population............: 141416
   Number of cases.......: 94606
   Expected cases........: 85032.80
   Observed / expected...: 1.11
   Relative risk.........: 1.12
   Percent cases in area.: 66.9
   Log likelihood ratio..: 1427.789132
   Monte Carlo rank......: 1/1000
   P-value...............: 0.001

6. Location IDs included.: 100440, 100420, 100410, 100430, 100460
   Coordinates / radius..: (49.355298 N, 6.775427 E) / 29.75 km
   Population............: 61262
   Number of cases.......: 30707
   Expected cases........: 36836.56
   Observed / expected...: 0.83
   Relative risk.........: 0.83
   Percent cases in area.: 50.1
   Log likelihood ratio..: 1270.805666
   Monte Carlo rank......: 1/1000
   P-value...............: 0.001

7. Location IDs included.: 40120, 33520, 34610, 33560, 34550, 40110, 34030, 34510, 33590, 34620, 33570, 34580, 10510, 33610, 10610, 10560, 34570, 34520, 34530, 32510, 20000, 33530, 34600, 33580, 32560, 10600, 10580, 10620, 34540, 10540, 33550, 57700, 10530, 34590
   Coordinates / radius..: (53.542867 N, 8.576513 E) / 133.89 km
   Population............: 392262
   Number of cases.......: 223428
   Expected cases........: 235865.35
   Observed / expected...: 0.95
   Relative risk.........: 0.94
   Percent cases in area.: 57.0
   Log likelihood ratio..: 896.607212
   Monte Carlo rank......: 1/1000
   P-value...............: 0.001

Supplementary file 2

8. Location IDs included.: 73150, 64140, 73390, 64330, 64360, 64390, 73310, 64110
Coordinates / radius..: (49.974177 N, 8.241514 E) / 31.98 km
Population............: 71887
Number of cases.......: 39273
Expected cases........: 43225.33
Observed / expected...: 0.91
Relative risk.........: 0.91
Percent cases in area.: 54.6
Log likelihood ratio..: 454.795093
Monte Carlo rank......: 1/1000
P-value...............: 0.001

9. Location IDs included.: 55540, 55580, 55620, 51700, 55120, 55130, 55150
Coordinates / radius..: (51.961076 N, 6.899097 E) / 49.62 km
Population............: 95062
Number of cases.......: 53214
Expected cases........: 57160.35
Observed / expected...: 0.93
Relative risk.........: 0.93
Percent cases in area.: 56.0
Log likelihood ratio..: 345.714083
Monte Carlo rank......: 1/1000
P-value...............: 0.001

10. Location IDs included.: 59700, 59660, 65320, 71320, 65340, 59580, 53740
Coordinates / radius..: (50.937659 N, 8.194734 E) / 48.15 km
Population............: 74261
Number of cases.......: 41353
Expected cases........: 44652.80
Observed / expected...: 0.93
Relative risk.........: 0.92
Percent cases in area.: 55.7
Log likelihood ratio..: 307.718816
Monte Carlo rank......: 1/1000
P-value...............: 0.001

11. Location IDs included.: 53620, 53150, 53580, 53160, 51620, 53140, 53780, 51160, 51110
Coordinates / radius..: (50.904871 N, 6.716684 E) / 37.31 km
Population............: 205435
Number of cases.......: 120299
Expected cases........: 123527.13
Observed / expected...: 0.97
Relative risk.........: 0.97
Percent cases in area.: 58.6
Log likelihood ratio..: 110.545535
Monte Carlo rank......: 1/1000
P-value...............: 0.001

Supplementary file 2

12. Location IDs included.: 59140, 59540, 59620, 59130, 59110, 51240, 51200, 59780, 59160, 51130, 51220
    Coordinates / radius..: (51.348029 N, 7.497563 E) / 36.41 km
    Population............: 201707
    Number of cases.......: 124156
    Expected cases........: 121285.50
    Observed / expected...: 1.02
    Relative risk.........: 1.02
    Percent cases in area.: 61.6
    Log likelihood ratio..: 89.658819
    Monte Carlo rank......: 1/1000
    P-value...............: 0.001

13. Location IDs included.: 57740
    Coordinates / radius..: (51.664034 N, 8.719670 E) / 0 km
    Population............: 17805
    Number of cases.......: 10003
    Expected cases........: 10706.07
    Observed / expected...: 0.93
    Relative risk.........: 0.93
    Percent cases in area.: 56.2
    Log likelihood ratio..: 57.563240
    Monte Carlo rank......: 1/1000
    P-value...............: 0.001

14. Location IDs included.: 51140, 51120, 51660
    Coordinates / radius..: (51.345236 N, 6.579665 E) / 18.94 km
    Population............: 47701
    Number of cases.......: 29723
    Expected cases........: 28682.39
    Observed / expected...: 1.04
    Relative risk.........: 1.04
    Percent cases in area.: 62.3
    Log likelihood ratio..: 48.175398
    Monte Carlo rank......: 1/1000
    P-value...............: 0.001
