# Supplementary material for: Regional variations in multimorbidity burden among office-based physicians in Germany
Source: Eur J Public Health. 2023 Mar 15;33(3):389–95. doi: 10.1093/eurpub/ckad039 (PMC10234650; doi:10.1093/eurpub/ckad039)
Supplement: ckad039_Supplementary_Data [file ckad039_supplementary_data.zip › ckad039_Supplementary_Data/ejph-2022-10-om-0501-File008.pdf]

	A	B	C	D	E	F	G	H	I	J
1	**State**	**Region (MB)**	**Cluster GP**	**Low rate**	**High rate**	**Average cluster supply**	**Moran's I p-value**	**Moran's I q-value**	**Overlap high**	**Overlap low**
2	Brandenburg	1203040	1		1	2.87	0.002	1	1	
3	Brandenburg	1204010	1		1	2.87	0.002	1	1	
4	Brandenburg	1204020	1		1	2.87	0.002	1	1	
5	Brandenburg	1204030	1		1	2.87	0.002	1	1	
6	Brandenburg	1204040	1		1	2.87	0.002	1	1	
7	Brandenburg	1204050	1		1	2.87	0.002	1	1	
8	Brandenburg	1204060	1		1	2.87	0.002	1	1	
9	Brandenburg	1204070	1		1	2.87	0.004	1	1	
10	Brandenburg	1204080	1		1	2.87	0.002	1	1	
11	Brandenburg	1204090	1		1	2.87	0.002	1	1	
12	Brandenburg	1204100	1		1	2.87	0.002	1	1	
13	Brandenburg	1204110	1		1	2.87	0.002	1	1	
14	Brandenburg	1204120	1		1	2.87	0.002	1	1	
15	Brandenburg	1204130	1		1	2.87	0.048	1	1	
16	Brandenburg	1205030	1		1	2.87	0.026	1	1	
17	Brandenburg	1205040	1		1	2.87				
18	Brandenburg	1205050	1		1	2.87	0.002	1	1	
19	Brandenburg	1205070	1		1	2.87	0.01	1	1	
20	Brandenburg	1205080	1		1	2.87	0.004	1	1	
21	Brandenburg	1205100	1		1	2.87				
22	Brandenburg	1205110	1		1	2.87	0.002	1	1	
23	Brandenburg	1205120	1		1	2.87	0.016	1	1	
24	Brandenburg	1205130	1		1	2.87	0.002	1	1	
25	Saxony-Anhalt	1502020	1		1	2.87	0.002	1	1	
26	Saxony-Anhalt	1502050	1		1	2.87	0.002	1	1	
27	Saxony-Anhalt	1503010	1		1	2.87	0.002	1	1	
28	Saxony-Anhalt	1503020	1		1	2.87	0.002	1	1	
29	Saxony-Anhalt	1503030	1		1	2.87	0.002	1	1	
30	Saxony-Anhalt	1503040	1		1	2.87	0.002	1	1	
31	Saxony-Anhalt	1503050	1		1	2.87	0.002	1	1	
32	Saxony-Anhalt	1503060	1		1	2.87	0.002	1	1	
33	Saxony-Anhalt	1503070	1		1	2.87	0.004	1	1	
34	Saxony-Anhalt	1504010	1		1	2.87	0.002	1	1	
35	Saxony-Anhalt	1504020	1		1	2.87	0.002	1	1	
36	Saxony-Anhalt	1504030	1		1	2.87	0.004	1	1	
37	Saxony-Anhalt	1504040	1		1	2.87	0.002	1	1	
38	Saxony-Anhalt	1504060	1		1	2.87	0.002	1	1	
39	Saxony-Anhalt	1505040	1		1	2.87	0.002	1	1	
40	Saxony-Anhalt	1505050	1		1	2.87	0.004	1	1	
41	Saxony-Anhalt	1505060	1		1	2.87	0.004	1	1	
42	Thuringia	1600200	1		1	2.87	0.002	1	1	
43	Thuringia	1601900	1		1	2.87	0.002	1	1	
44	Thuringia	1602000	1		1	2.87	0.008	1	1	
45	Thuringia	1602500	1		1	2.87	0.004	1	1	
46	Thuringia	1602600	1		1	2.87	0.002	1	1	
47	Thuringia	1602700	1		1	2.87	0.004	1	1	
48	Thuringia	1602900	1		1	2.87	0.002	1	1	
49	Thuringia	1603000	1		1	2.87	0.002	1	1	
50	Thuringia	1603100	1		1	2.87	0.002	1	1	
51	Thuringia	1603200	1		1	2.87	0.002	1	1	
52	Thuringia	1603400	1		1	2.87	0.004	1	1	
53	Thuringia	1603500	1		1	2.87	0.002	1	1	
54	Saxony	3400164	1		1	2.87	0.004	1	1	
55	Saxony	3400165	1		1	2.87	0.002	1	1	
56	Saxony	3400166	1		1	2.87	0.01	1	1	
57	Saxony	3400167	1		1	2.87	0.01	1	1	
58	Saxony	3400168	1		1	2.87	0.04	1	1	
59	Saxony	3400169	1		1	2.87	0.044	1	1	
60	Saxony	3400170	1		1	2.87				
61	Saxony	3400171	1		1	2.87	0.012	1	1	
62	Saxony	3400172	1		1	2.87	0.024	1	1	
63	Saxony	3400173	1		1	2.87	0.006	1	1	
64	Saxony	3400174	1		1	2.87				
65	Saxony	3400176	1		1	2.87	0.002	1	1	

	A	B	C	D	E	F	G	H	I	J
1	**State**	**Region (MB)**	**Cluster GP**	**Low rate**	**High rate**	**Average cluster supply**	**Moran's I p-value**	**Moran's I q-value**	**Overlap high**	**Overlap low**
66	Saxony	3400177	1		1	2.87	0.004	1	1	
67	Saxony	3400178	1		1	2.87	0.004	1	1	
68	Saxony	3400179	1		1	2.87	0.006	1	1	
69	Saxony	3400180	1		1	2.87				
70	Saxony	3400181	1		1	2.87	0.002	1	1	
71	Saxony	3400182	1		1	2.87	0.004	1	1	
72	Saxony	3400183	1		1	2.87	0.002	1	1	
73	Saxony	3400184	1		1	2.87	0.006	1	1	
74	Saxony	3400185	1		1	2.87	0.014	1	1	
75	Saxony	3400186	1		1	2.87	0.002	1	1	
76	Saxony	3400187	1		1	2.87	0.002	1	1	
77	Saxony	3400188	1		1	2.87	0.004	1	1	
78	Saxony	3400189	1		1	2.87	0.016	1	1	
79	Saxony	3400190	1		1	2.87	0.01	1	1	
80	Saxony	3400191	1		1	2.87	0.044	1	1	
81	Saxony	3400192	1		1	2.87				
82	Saxony	3400193	1		1	2.87	0.032	1	1	
83	Saxony	3400194	1		1	2.87	0.006	1	1	
84	Saxony	3400196	1		1	2.87	0.006	1	1	
85	Saxony	3400197	1		1	2.87	0.022	1	1	
86	Saxony	3400198	1		1	2.87	0.032	1	1	
87	Saxony	3400199	1		1	2.87	0.008	1	1	
88	Saxony	3400200	1		1	2.87	0.004	1	1	
89	Saxony	3400201	1		1	2.87	0.002	1	1	
90	Saxony	3400202	1		1	2.87	0.008	1	1	
91	Saxony	3400203	1		1	2.87	0.002	1	1	
92	Saxony	3400204	1		1	2.87	0.022	1	1	
93	Saxony	3400206	1		1	2.87	0.004	1	1	
94	Saxony	3400207	1		1	2.87	0.002	1	1	
95	Saxony	3400208	1		1	2.87	0.006	1	1	
96	Saxony	3400209	1		1	2.87	0.002	1	1	
97	Saxony	3400321	1		1	2.87	0.004	1	1	
98	Saxony	3400407	1		1	2.87	0.012	1	1	
99	Saxony	3400408	1		1	2.87	0.006	1	1	
100	Saxony	3400409	1		1	2.87	0.002	1	1	
101	Saxony-Anhalt	3500159	1		1	2.87	0.002	1	1	
102	Saxony-Anhalt	3500161	1		1	2.87				
103	Saxony-Anhalt	3500162	1		1	2.87	0.018	1	1	
104	Thuringia	3600522	1		1	2.87	0.002	1	1	
105	Thuringia	3600524	1		1	2.87	0.008	1	1	
106	Thuringia	3600526	1		1	2.87	0.002	1	1	
107	Thuringia	3600527	1		1	2.87	0.004	1	1	
108	Thuringia	3600528	1		1	2.87	0.006	1	1	
109	Baden-Württemberg	801010	2	1		3.07				
110	Baden-Württemberg	801020	2	1		3.07	0.02	3		1
111	Baden-Württemberg	801030	2	1		3.07	0.004	3		1
112	Baden-Württemberg	801040	2	1		3.07	0.008	3		1
113	Baden-Württemberg	801050	2	1		3.07	0.002	3		1
114	Baden-Württemberg	801060	2	1		3.07	0.012	3		1
115	Baden-Württemberg	801070	2	1		3.07	0.008	3		1
116	Baden-Württemberg	801080	2	1		3.07	0.002	3		1
117	Baden-Württemberg	801090	2	1		3.07	0.018	3		1
118	Baden-Württemberg	801100	2	1		3.07	0.006	3		1
119	Baden-Württemberg	801110	2	1		3.07	0.002	3		1
120	Baden-Württemberg	801120	2	1		3.07	0.002	3		1
121	Baden-Württemberg	801130	2	1		3.07	0.006	3		1
122	Baden-Württemberg	801140	2	1		3.07	0.006	3		1
123	Baden-Württemberg	801150	2	1		3.07	0.002	3		1
124	Baden-Württemberg	802050	2	1		3.07	0.01	3		1
125	Baden-Württemberg	802080	2	1		3.07	0.006	3		1
126	Baden-Württemberg	802090	2	1		3.07	0.002	3		1
127	Baden-Württemberg	803010	2	1		3.07	0.004	3		1
128	Baden-Württemberg	803020	2	1		3.07	0.01	3		1
129	Baden-Württemberg	803030	2	1		3.07	0.006	3		1

	A	B	C	D	E	F	G	H	I	J
1	**State**	**Region (MB)**	**Cluster GP**	**Low rate**	**High rate**	**Average cluster supply**	**Moran's I p-value**	**Moran's I q-value**	**Overlap high**	**Overlap low**
130	Baden-Württemberg	803040	2	1		3.07	0.01	3		1
131	Baden-Württemberg	806040	2	1		3.07	0.018	3		1
132	Baden-Württemberg	806050	2	1		3.07	0.002	3		1
133	Baden-Württemberg	806060	2	1		3.07	0.002	3		1
134	Baden-Württemberg	806070	2	1		3.07	0.002	3		1
135	Baden-Württemberg	808010	2	1		3.07	0.044	3		1
136	Baden-Württemberg	808020	2	1		3.07	0.002	3		1
137	Baden-Württemberg	808040	2	1		3.07	0.014	3		1
138	Baden-Württemberg	808050	2	1		3.07	0.006	3		1
139	Baden-Württemberg	809060	2	1		3.07	0.018	3		1
140	Baden-Württemberg	809070	2	1		3.07	0.032	3		1
141	Baden-Württemberg	809080	2	1		3.07	0.038	3		1
142	Baden-Württemberg	809090	2	1		3.07				
143	Baden-Württemberg	810010	2	1		3.07	0.002	3		1
144	Baden-Württemberg	810020	2	1		3.07	0.002	3		1
145	Baden-Württemberg	810030	2	1		3.07	0.002	3		1
146	Baden-Württemberg	810040	2	1		3.07	0.002	3		1
147	Baden-Württemberg	810050	2	1		3.07	0.002	3		1
148	Baden-Württemberg	810060	2	1		3.07	0.002	3		1
149	Baden-Württemberg	810070	2	1		3.07	0.004	3		1
150	Baden-Württemberg	810080	2	1		3.07	0.006	4		1
151	Baden-Württemberg	811010	2	1		3.07	0.002	3		1
152	Baden-Württemberg	811020	2	1		3.07	0.002	3		1
153	Baden-Württemberg	811030	2	1		3.07	0.002	3		1
154	Baden-Württemberg	811040	2	1		3.07	0.002	3		1
155	Baden-Württemberg	811050	2	1		3.07	0.008	3		1
156	Baden-Württemberg	811060	2	1		3.07	0.002	3		1
157	Baden-Württemberg	812010	2	1		3.07	0.002	3		1
158	Baden-Württemberg	812020	2	1		3.07	0.006	3		1
159	Baden-Württemberg	812030	2	1		3.07	0.02	3		1
160	Baden-Württemberg	812040	2	1		3.07	0.004	3		1
161	Baden-Württemberg	812050	2	1		3.07				
162	Baden-Württemberg	812060	2	1		3.07				
163	Baden-Württemberg	812070	2	1		3.07	0.018	3		1
164	Baden-Württemberg	812080	2	1		3.07	0.012	3		1
165	Baden-Württemberg	812090	2	1		3.07	0.002	3		1
166	Bavaria	909040	2	1		3.07	0.002	3		1
167	Bavaria	909050	2	1		3.07	0.01	3		1
168	Bavaria	910030	2	1		3.07	0.002	3		1
169	Bavaria	910040	2	1		3.07	0.002	3		1
170	Bavaria	914030	2	1		3.07	0.002	3		1
171	Bavaria	914050	2	1		3.07	0.004	3		1
172	Bavaria	914080	2	1		3.07	0.002	3		1
173	Bavaria	915010	2	1		3.07	0.004	3		1
174	Bavaria	915020	2	1		3.07				
175	Bavaria	915030	2	1		3.07	0.006	3		1
176	Bavaria	915040	2	1		3.07				
177	Bavaria	915050	2	1		3.07	0.006	4		
178	Bavaria	915060	2	1		3.07				
179	Bavaria	916010	2	1		3.07	0.004	3		1
180	Bavaria	916020	2	1		3.07	0.024	3		1
181	Bavaria	916030	2	1		3.07	0.002	3		1
182	Bavaria	916040	2	1		3.07	0.008	3		1
183	Bavaria	916050	2	1		3.07	0.042	3		1
184	Bavaria	916060	2	1		3.07	0.032	3		1
185	Bavaria	916070	2	1		3.07	0.038	3		1
186	Bavaria	916080	2	1		3.07				
187	Bavaria	917010	2	1		3.07	0.002	3		1
188	Bavaria	917020	2	1		3.07	0.004	3		1
189	Bavaria	917040	2	1		3.07	0.012	3		1
190	Bavaria	917080	2	1		3.07	0.002	3		1
191	Bavaria	2900122	2	1		3.07	0.004	3		1
192	Bavaria	2900123	2	1		3.07				
193	Bavaria	2900124	2	1		3.07	0.008	3		1

	A	B	C	D	E	F	G	H	I	J
1	**State**	**Region (MB)**	**Cluster GP**	**Low rate**	**High rate**	**Average cluster supply**	**Moran's I p-value**	**Moran's I q-value**	**Overlap high**	**Overlap low**
194	Bavaria	2900132	2	1		3.07				
195	Bavaria	2900142	2	1		3.07	0.01	3		1
196	Bavaria	2900143	2	1		3.07				
197	Bavaria	2900144	2	1		3.07	0.004	3		1
198	Bavaria	2900145	2	1		3.07	0.016	3		1
199	Bavaria	2900152	2	1		3.07				
200	Bavaria	2900302	2	1		3.07				
201	Bavaria	2900303	2	1		3.07	0.024	3		1
202	Bavaria	2900309	2	1		3.07	0.022	3		1
203	Bavaria	2900310	2	1		3.07	0.046	3		1
204	Bavaria	2900343	2	1		3.07	0.014	3		1
205	Bavaria	2900352	2	1		3.07	0.002	3		1
206	Bavaria	2900371	2	1		3.07	0.016	3		1
207	Bavaria	2900373	2	1		3.07	0.002	3		1
208	Bavaria	2900391	2	1		3.07	0.036	3		1
209	Brandenburg	1201010	3		1	2.77	0.002	1	1	
210	Brandenburg	1201030	3		1	2.77				
211	Brandenburg	1201040	3		1	2.77	0.026	1	1	
212	Brandenburg	1201050	3		1	2.77	0.006	1	1	
213	Brandenburg	1201060	3		1	2.77	0.016	1	1	
214	Brandenburg	1201070	3		1	2.77				
215	Brandenburg	1201080	3		1	2.77	0.01	1	1	
216	Brandenburg	1202010	3		1	2.77	0.002	1	1	
217	Brandenburg	1202020	3		1	2.77	0.002	1	1	
218	Brandenburg	1202030	3		1	2.77	0.002	1	1	
219	Brandenburg	1202040	3		1	2.77	0.004	1	1	
220	Brandenburg	1202050	3		1	2.77	0.002	1	1	
221	Brandenburg	1203060	3		1	2.77	0.004	1	1	
222	Mecklenburg-Western Pomerania	1301060	3		1	2.77	0.002	1	1	
223	Mecklenburg-Western Pomerania	1302040	3		1	2.77	0.002	1	1	
224	Mecklenburg-Western Pomerania	1302050	3		1	2.77	0.004	1	1	
225	Mecklenburg-Western Pomerania	1303070	3		1	2.77	0.002	1	1	
226	Mecklenburg-Western Pomerania	1303080	3		1	2.77	0.002	1	1	
227	Mecklenburg-Western Pomerania	3300155	3		1	2.77	0.004	1	1	
228	Mecklenburg-Western Pomerania	3300501	3		1	2.77	0.002	1	1	
229	Mecklenburg-Western Pomerania	3300502	3		1	2.77	0.002	1	1	
230	Mecklenburg-Western Pomerania	3300503	3		1	2.77				
231	Mecklenburg-Western Pomerania	3300504	3		1	2.77				
232	Mecklenburg-Western Pomerania	3300505	3		1	2.77				
233	Mecklenburg-Western Pomerania	3300506	3		1	2.77	0.006	1	1	
234	Mecklenburg-Western Pomerania	3300507	3		1	2.77	0.002	1	1	
235	Mecklenburg-Western Pomerania	3300508	3		1	2.77	0.014	1	1	
236	Mecklenburg-Western Pomerania	3300509	3		1	2.77	0.002	1	1	
237	Mecklenburg-Western Pomerania	3300510	3		1	2.77				
238	Mecklenburg-Western Pomerania	3300511	3		1	2.77	0.002	1	1	
239	Mecklenburg-Western Pomerania	3300512	3		1	2.77	0.012	1	1	
240	Mecklenburg-Western Pomerania	3300513	3		1	2.77				
241	Mecklenburg-Western Pomerania	3300514	3		1	2.77	0.002	1	1	
242	Mecklenburg-Western Pomerania	3300515	3		1	2.77	0.002	1	1	
243	Mecklenburg-Western Pomerania	3300516	3		1	2.77	0.002	1	1	
244	Mecklenburg-Western Pomerania	3300517	3		1	2.77				
245	Mecklenburg-Western Pomerania	3300518	3		1	2.77	0.004	1	1	
246	Mecklenburg-Western Pomerania	3300519	3		1	2.77				
247	Mecklenburg-Western Pomerania	3300520	3		1	2.77				
248	Schleswig-Holstein	101090	4	1		2.97				
249	Schleswig-Holstein	101110	4	1		2.97				
250	Schleswig-Holstein	101120	4	1		2.97				
251	Schleswig-Holstein	103010	4	1		2.97				
252	Schleswig-Holstein	103020	4	1		2.97	0.048	3		1
253	Schleswig-Holstein	103040	4	1		2.97				
254	Schleswig-Holstein	104010	4	1		2.97				
255	Schleswig-Holstein	104020	4	1		2.97				
256	Schleswig-Holstein	104030	4	1		2.97				
257	Schleswig-Holstein	104040	4	1		2.97	0.046	3		1

	A	B	C	D	E	F	G	H	I	J
1	**State**	**Region (MB)**	**Cluster GP**	**Low rate**	**High rate**	**Average cluster supply**	**Moran's I p-value**	**Moran's I q-value**	**Overlap high**	**Overlap low**
258	Schleswig-Holstein	105050	4	1		2.97				
259	Schleswig-Holstein	105070	4	1		2.97				
260	Lower Saxony	306010	4	1		2.97				
261	Lower Saxony	306030	4	1		2.97				
262	Lower Saxony	307020	4	1		2.97				
263	Lower Saxony	307030	4	1		2.97				
264	Lower Saxony	314020	4	1		2.97				
265	Lower Saxony	315010	4	1		2.97	0.002	3		1
266	Lower Saxony	319010	4	1		2.97	0.016	3		1
267	Lower Saxony	319020	4	1		2.97	0.02	3		1
268	Lower Saxony	319030	4	1		2.97	0.022	3		1
269	Lower Saxony	320010	4	1		2.97	0.03	3		1
270	Lower Saxony	320020	4	1		2.97				
271	Lower Saxony	320030	4	1		2.97				
272	Lower Saxony	321010	4	1		2.97	0.028	3		1
273	Lower Saxony	321020	4	1		2.97	0.002	3		1
274	Lower Saxony	323020	4	1		2.97				
275	Lower Saxony	325010	4	1		2.97				
276	Lower Saxony	329010	4	1		2.97				
277	Lower Saxony	330010	4	1		2.97				
278	Lower Saxony	330020	4	1		2.97				
279	Lower Saxony	331010	4	1		2.97				
280	Lower Saxony	331020	4	1		2.97	0.012	3		1
281	Lower Saxony	332010	4	1		2.97				
282	Lower Saxony	332020	4	1		2.97	0.048	3		1
283	Lower Saxony	333020	4	1		2.97				
284	Lower Saxony	337010	4	1		2.97	0.018	3		1
285	Lower Saxony	337020	4	1		2.97	0.038	3		1
286	Lower Saxony	338010	4	1		2.97	0.018	3		1
287	Lower Saxony	338020	4	1		2.97	0.014	3		1
288	Lower Saxony	339010	4	1		2.97				
289	Nordrhein-Westfalen	504010	4	1		2.97				
290	Nordrhein-Westfalen	504020	4	1		2.97				
291	Schleswig-Holstein	2100080	4	1		2.97				
292	Schleswig-Holstein	2100082	4	1		2.97				
293	Schleswig-Holstein	2100083	4	1		2.97	0.014	3		1
294	Schleswig-Holstein	2100084	4	1		2.97	0.028	3		1
295	Hamburg	2200000	4	1		2.97	0.014	3		1
296	Lower Saxony	2300004	4	1		2.97				
297	Lower Saxony	2300085	4	1		2.97	0.018	3		1
298	Lower Saxony	2300086	4	1		2.97				
299	Lower Saxony	2300087	4	1		2.97				
300	Lower Saxony	2300090	4	1		2.97	0.034	4		
301	Lower Saxony	2300096	4	1		2.97	0.024	3		1
302	Lower Saxony	2300099	4	1		2.97				
303	Lower Saxony	2300100	4	1		2.97				
304	Lower Saxony	2300101	4	1		2.97				
305	Lower Saxony	2300102	4	1		2.97	0.028	3		1
306	Lower Saxony	2300103	4	1		2.97	0.02	3		1
307	Lower Saxony	2300105	4	1		2.97				
308	Lower Saxony	2300107	4	1		2.97				
309	Lower Saxony	2300108	4	1		2.97				
310	Lower Saxony	2300111	4	1		2.97				
311	Lower Saxony	2300112	4	1		2.97				
312	Lower Saxony	2300113	4	1		2.97	0.008	3		1
313	Lower Saxony	2300114	4	1		2.97	0.02	3		1
314	Lower Saxony	2300115	4	1		2.97				
315	Lower Saxony	2300429	4	1		2.97				
316	Lower Saxony	2300432	4	1		2.97				
317	Lower Saxony	2300434	4	1		2.97				
318	Lower Saxony	2300437	4	1		2.97				
319	Lower Saxony	2300441	4	1		2.97				
320	Lower Saxony	2300442	4	1		2.97	0.042	3		1
321	Bremen	2400000	4	1		2.97				

	A	B	C	D	E	F	G	H	I	J
1	**State**	**Region (MB)**	**Cluster GP**	**Low rate**	**High rate**	**Average cluster supply**	**Moran's I p-value**	**Moran's I q-value**	**Overlap high**	**Overlap low**
322	Bremen	2400001	4	1		2.97				
323	Hessen	601050	5		1	3.46				
324	Hessen	601090	5		1	3.46				
325	Hessen	601100	5		1	3.46				
326	Hessen	601120	5		1	3.46				
327	Hessen	601130	5		1	3.46				
328	Hessen	601150	5		1	3.46				
329	Hessen	601170	5		1	3.46				
330	Hessen	601180	5		1	3.46				
331	Hessen	601190	5		1	3.46				
332	Hessen	601200	5		1	3.46				
333	Hessen	601210	5		1	3.46				
334	Hessen	601220	5		1	3.46				
335	Hessen	602060	5		1	3.46				
336	Hessen	602070	5		1	3.46				
337	Hessen	602080	5		1	3.46				
338	Hessen	602120	5		1	3.46				
339	Hessen	603060	5		1	3.46				
340	Hessen	603070	5		1	3.46				
341	Hessen	603090	5		1	3.46				
342	Bavaria	903020	5		1	3.46				
343	Bavaria	903030	5		1	3.46				
344	Bavaria	903040	5		1	3.46				
345	Bavaria	904010	5		1	3.46				
346	Bavaria	904020	5		1	3.46				
347	Thuringia	1600300	5		1	3.46	0.002	1	1	
348	Thuringia	1600400	5		1	3.46	0.012	1	1	
349	Thuringia	1600500	5		1	3.46	0.004	1	1	
350	Thuringia	1600600	5		1	3.46	0.044	1	1	
351	Thuringia	1600700	5		1	3.46	0.014	1	1	
352	Thuringia	1600800	5		1	3.46	0.008	1	1	
353	Thuringia	1600900	5		1	3.46				
354	Thuringia	1601000	5		1	3.46	0.026	1	1	
355	Thuringia	1601100	5		1	3.46	0.004	1	1	
356	Thuringia	1601200	5		1	3.46	0.002	1	1	
357	Thuringia	1601300	5		1	3.46	0.002	1	1	
358	Thuringia	1601500	5		1	3.46	0.004	1	1	
359	Thuringia	1601800	5		1	3.46	0.004	1	1	
360	Thuringia	1602200	5		1	3.46	0.002	1	1	
361	Thuringia	1602300	5		1	3.46	0.006	1	1	
362	Lower Saxony	2300095	5		1	3.46				
363	Hessen	2600270	5		1	3.46				
364	Hessen	2600271	5		1	3.46				
365	Hessen	2600272	5		1	3.46				
366	Hessen	2600273	5		1	3.46				
367	Hessen	2600274	5		1	3.46				
368	Bavaria	2900127	5		1	3.46				
369	Bavaria	2900148	5		1	3.46				
370	Bavaria	2900149	5		1	3.46				
371	Bavaria	2900304	5		1	3.46				
372	Bavaria	2900345	5		1	3.46				
373	Bavaria	2900346	5		1	3.46				
374	Bavaria	2900372	5		1	3.46				
375	Thuringia	3600521	5		1	3.46	0.032	1	1	
376	Thuringia	3600523	5		1	3.46	0.002	1	1	
377	Hessen	602150	6	1		3.32				
378	Hessen	603020	6	1		3.32				
379	Hessen	603100	6	1		3.32				
380	Hessen	603110	6	1		3.32				
381	Hessen	603120	6	1		3.32				
382	Hessen	603130	6	1		3.32				
383	Hessen	603140	6	1		3.32				
384	Hessen	603150	6	1		3.32				
385	Hessen	603160	6	1		3.32				

	A	B	C	D	E	F	G	H	I	J
1	**State**	**Region (MB)**	**Cluster GP**	**Low rate**	**High rate**	**Average cluster supply**	**Moran's I p-value**	**Moran's I q-value**	**Overlap high**	**Overlap low**
386	Hessen	603170	6	1		3.32				
387	Hessen	603180	6	1		3.32				
388	Hessen	603190	6	1		3.32				
389	Hessen	603200	6	1		3.32				
390	Hessen	603250	6	1		3.32				
391	Hessen	603260	6	1		3.32	0.032	3		1
392	Hessen	603270	6	1		3.32				
393	Rhineland-Palatinate	701110	6	1		3.32				
394	Rhineland-Palatinate	703010	6	1		3.32				
395	Rhineland-Palatinate	703020	6	1		3.32	0.038	3		1
396	Rhineland-Palatinate	703030	6	1		3.32	0.046	3		1
397	Rhineland-Palatinate	703050	6	1		3.32				
398	Rhineland-Palatinate	2700116	6	1		3.32				
399	Lower Saxony	332030	7	1		3.18				
400	Nordrhein-Westfalen	503010	7	1		3.18	0.046	3		1
401	Nordrhein-Westfalen	503020	7	1		3.18				
402	Nordrhein-Westfalen	503040	7	1		3.18	0.034	3		1
403	Nordrhein-Westfalen	503050	7	1		3.18				
404	Nordrhein-Westfalen	503060	7	1		3.18	0.05	3		1
405	Nordrhein-Westfalen	503070	7	1		3.18	0.008	3		1
406	Nordrhein-Westfalen	503080	7	1		3.18				
407	Nordrhein-Westfalen	503090	7	1		3.18				
408	Nordrhein-Westfalen	503100	7	1		3.18				
409	Nordrhein-Westfalen	503110	7	1		3.18				
410	Nordrhein-Westfalen	503120	7	1		3.18				
411	Nordrhein-Westfalen	503130	7	1		3.18	0.014	3		1
412	Nordrhein-Westfalen	503140	7	1		3.18				
413	Nordrhein-Westfalen	503150	7	1		3.18				
414	Nordrhein-Westfalen	503160	7	1		3.18				
415	Nordrhein-Westfalen	503170	7	1		3.18				
416	Nordrhein-Westfalen	503180	7	1		3.18				
417	Nordrhein-Westfalen	503230	7	1		3.18				
418	Nordrhein-Westfalen	503240	7	1		3.18				
419	Nordrhein-Westfalen	503270	7	1		3.18				
420	Lower Saxony	2300438	7	1		3.18				
421	Bavaria	905040	8	1		3.40				
422	Bavaria	905080	8	1		3.40				
423	Bavaria	906010	8	1		3.40				
424	Bavaria	906020	8	1		3.40				
425	Bavaria	906040	8	1		3.40				
426	Bavaria	906070	8	1		3.40				
427	Bavaria	907050	8	1		3.40				
428	Bavaria	907060	8	1		3.40				
429	Bavaria	911010	8	1		3.40				
430	Bavaria	911030	8	1		3.40				
431	Bavaria	911050	8	1		3.40				
432	Bavaria	911060	8	1		3.40				
433	Bavaria	911070	8	1		3.40	0.014	3		1
434	Bavaria	911080	8	1		3.40	0.022	3		1
435	Bavaria	911090	8	1		3.40				
436	Bavaria	912010	8	1		3.40				
437	Bavaria	912050	8	1		3.40				
438	Bavaria	912060	8	1		3.40				
439	Bavaria	912080	8	1		3.40				
440	Bavaria	912090	8	1		3.40				
441	Bavaria	912100	8	1		3.40				
442	Bavaria	913010	8	1		3.40	0.002	3		1
443	Bavaria	913030	8	1		3.40				
444	Bavaria	913070	8	1		3.40				
445	Bavaria	913080	8	1		3.40				
446	Bavaria	914010	8	1		3.40	0.004	3		1
447	Bavaria	918010	8	1		3.40	0.004	3		1
448	Bavaria	918020	8	1		3.40				
449	Bavaria	2900128	8	1		3.40				

	A	B	C	D	E	F	G	H	I	J
1	**State**	**Region (MB)**	**Cluster GP**	**Low rate**	**High rate**	**Average cluster supply**	**Moran's I p-value**	**Moran's I q-value**	**Overlap high**	**Overlap low**
450	Bavaria	2900129	8	1		3.40				
451	Bavaria	2900130	8	1		3.40	0.002	3		1
452	Bavaria	2900135	8	1		3.40				
453	Bavaria	2900136	8	1		3.40	0.002	3		1
454	Bavaria	2900137	8	1		3.40	0.006	3		1
455	Bavaria	2900138	8	1		3.40	0.002	3		1
456	Bavaria	2900141	8	1		3.40				
457	Bavaria	2900146	8	1		3.40				
458	Bavaria	2900341	8	1		3.40				
459	Bavaria	2900342	8	1		3.40	0.004	3		1
460	Bavaria	2900344	8	1		3.40	0.032	3		1
461	Bavaria	2900348	8	1		3.40				
462	Bavaria	2900350	8	1		3.40				
463	Bavaria	2900351	8	1		3.40				
464	Bavaria	2900353	8	1		3.40	0.036	3		1
465	Bavaria	2900356	8	1		3.40				
466	Bavaria	2900357	8	1		3.40				
467	Bavaria	2900358	8	1		3.40	0.016	3		1
468	Bavaria	2900359	8	1		3.40				
469	Bavaria	2900361	8	1		3.40				
470	Bavaria	2900362	8	1		3.40				
471	Bavaria	2900367	8	1		3.40				
472	Bavaria	2900368	8	1		3.40				
473	Bavaria	2900370	8	1		3.40				
474	Bavaria	2900375	8	1		3.40				
475	Bavaria	2900377	8	1		3.40				
476	Bavaria	2900379	8	1		3.40				
477	Bavaria	2900380	8	1		3.40				
478	Bavaria	2900383	8	1		3.40				
479	Bavaria	2900384	8	1		3.40				
480	Bavaria	2900385	8	1		3.40				
481	Bavaria	2900386	8	1		3.40				
482	Bavaria	2900392	8	1		3.40				
483	Bavaria	2900393	8	1		3.40				
484	Bavaria	2900394	8	1		3.40				
485	Bavaria	2900396	8	1		3.40				
486	Bavaria	2900397	8	1		3.40				
487	Bavaria	2900398	8	1		3.40				
488	Bavaria	2900399	8	1		3.40				
489	Bavaria	2900400	8	1		3.40				
490	Bavaria	2900401	8	1		3.40				
491	Bavaria	2900402	8	1		3.40				
492	Bavaria	2900406	8	1		3.40				
493	Nordrhein-Westfalen	501160	9		1	3.35				
494	Nordrhein-Westfalen	501170	9		1	3.35				
495	Nordrhein-Westfalen	501360	9		1	3.35				
496	Nordrhein-Westfalen	501370	9		1	3.35				
497	Nordrhein-Westfalen	501380	9		1	3.35				
498	Nordrhein-Westfalen	501390	9		1	3.35				
499	Nordrhein-Westfalen	501400	9		1	3.35				
500	Nordrhein-Westfalen	501410	9		1	3.35				
501	Nordrhein-Westfalen	501420	9		1	3.35				
502	Nordrhein-Westfalen	501430	9		1	3.35				
503	Nordrhein-Westfalen	501440	9		1	3.35				
504	Nordrhein-Westfalen	501450	9		1	3.35				
505	Nordrhein-Westfalen	501460	9		1	3.35				
506	Nordrhein-Westfalen	505320	9		1	3.35				
507	Nordrhein-Westfalen	505330	9		1	3.35				
508	Nordrhein-Westfalen	505340	9		1	3.35				
509	Nordrhein-Westfalen	505350	9		1	3.35				
510	Nordrhein-Westfalen	505360	9		1	3.35				
511	Nordrhein-Westfalen	505370	9		1	3.35				
512	Nordrhein-Westfalen	505390	9		1	3.35				
513	Rhineland-Palatinate	705020	10		1	3.33				

	A	B	C	D	E	F	G	H	I	J
1	**State**	**Region (MB)**	**Cluster GP**	**Low rate**	**High rate**	**Average cluster supply**	**Moran's I p-value**	**Moran's I q-value**	**Overlap high**	**Overlap low**
514	Rhineland-Palatinate	705030	10		1	3.33	0.028	1	1	
515	Rhineland-Palatinate	705040	10		1	3.33				
516	Rhineland-Palatinate	705050	10		1	3.33				
517	Rhineland-Palatinate	705060	10		1	3.33				
518	Saarland	1000080	10		1	3.33				
519	Nordrhein-Westfalen	502300	11	1		2.48	0.038	3		1
520	Nordrhein-Westfalen	502330	11	1		2.48				
521	Nordrhein-Westfalen	505070	11	1		2.48				
522	Nordrhein-Westfalen	505090	11	1		2.48				
523	Nordrhein-Westfalen	505120	11	1		2.48	0.01	3		1
524	Nordrhein-Westfalen	505130	11	1		2.48				
525	Nordrhein-Westfalen	505140	11	1		2.48	0.006	3		1
526	Nordrhein-Westfalen	505150	11	1		2.48				
527	Nordrhein-Westfalen	505160	11	1		2.48	0.01	3		1
528	Nordrhein-Westfalen	505170	11	1		2.48	0.016	3		1
529	Nordrhein-Westfalen	505180	11	1		2.48				
530	Nordrhein-Westfalen	505190	11	1		2.48	0.006	3		1
531	Nordrhein-Westfalen	505200	11	1		2.48				
532	Nordrhein-Westfalen	505210	11	1		2.48				
533	Nordrhein-Westfalen	505230	11	1		2.48				
534	Nordrhein-Westfalen	505240	11	1		2.48				
535	Hessen	601070	11	1		2.48				
536	Hessen	602010	11	1		2.48				
537	Hessen	602020	11	1		2.48				
538	Hessen	602090	11	1		2.48				
539	Rhineland-Palatinate	701010	11	1		2.48				
540	Nordrhein-Westfalen	504200	12	1		2.40				
541	Nordrhein-Westfalen	504210	12	1		2.40				
542	Nordrhein-Westfalen	504220	12	1		2.40				
543	Nordrhein-Westfalen	504230	12	1		2.40				
544	Nordrhein-Westfalen	505020	12	1		2.40				
545	Lower Saxony	306050	13		1	2.00				
546	Bavaria	902050	14	1		4.00				
547	Bavaria	903070	14	1		4.00				
548	Bavaria	2900313	14	1		4.00				
549	Bavaria	2900389	14	1		4.00				
550	Bavaria	2900403	14	1		4.00				
551	Bavaria	2900404	14	1		4.00				
552	Nordrhein-Westfalen	501230	15		1	2.92				
553	Nordrhein-Westfalen	501240	15		1	2.92				
554	Nordrhein-Westfalen	501250	15		1	2.92				
555	Nordrhein-Westfalen	501260	15		1	2.92				
556	Nordrhein-Westfalen	501270	15		1	2.92				
557	Nordrhein-Westfalen	501280	15		1	2.92				
558	Nordrhein-Westfalen	501290	15		1	2.92				
559	Nordrhein-Westfalen	501320	15		1	2.92				
560	Nordrhein-Westfalen	501330	15		1	2.92				
561	Nordrhein-Westfalen	502010	15		1	2.92				
562	Nordrhein-Westfalen	502020	15		1	2.92				
563	Nordrhein-Westfalen	502030	15		1	2.92				
564	Nordrhein-Westfalen	502040	15		1	2.92				
565	Nordrhein-Westfalen	502050	15		1	2.92				
566	Nordrhein-Westfalen	502060	15		1	2.92				
567	Nordrhein-Westfalen	502070	15		1	2.92				
568	Nordrhein-Westfalen	502080	15		1	2.92				
569	Nordrhein-Westfalen	502100	15		1	2.92				
570	Nordrhein-Westfalen	502110	15		1	2.92				
571	Nordrhein-Westfalen	502120	15		1	2.92				
572	Nordrhein-Westfalen	502130	15		1	2.92				
573	Nordrhein-Westfalen	502140	15		1	2.92				
574	Nordrhein-Westfalen	502150	15		1	2.92				
575	Nordrhein-Westfalen	502160	15		1	2.92				
576	Nordrhein-Westfalen	502170	15		1	2.92				
577	Nordrhein-Westfalen	502180	15		1	2.92				

	A	B	C	D	E	F	G	H	I	J
1	**State**	**Region (MB)**	**Cluster GP**	**Low rate**	**High rate**	**Average cluster supply**	**Moran's I p-value**	**Moran's I q-value**	**Overlap high**	**Overlap low**
578	Nordrhein-Westfalen	502190	15		1	2.92				
579	Nordrhein-Westfalen	502200	15		1	2.92				
580	Nordrhein-Westfalen	502220	15		1	2.92				
581	Nordrhein-Westfalen	502230	15		1	2.92				
582	Nordrhein-Westfalen	502240	15		1	2.92				
583	Nordrhein-Westfalen	502250	15		1	2.92				
584	Nordrhein-Westfalen	502260	15		1	2.92				
585	Nordrhein-Westfalen	502380	15		1	2.92				
586	Nordrhein-Westfalen	502390	15		1	2.92				
587	Nordrhein-Westfalen	502400	15		1	2.92				
588	Nordrhein-Westfalen	502410	15		1	2.92				
589	Nordrhein-Westfalen	502420	15		1	2.92				
590	Nordrhein-Westfalen	502430	16	1		4.00				
591	Nordrhein-Westfalen	503290	17		1	3.57				
592	Nordrhein-Westfalen	505400	17		1	3.57				
593	Nordrhein-Westfalen	505420	17		1	3.57				
594	Nordrhein-Westfalen	505430	17		1	3.57				
595	Nordrhein-Westfalen	505440	17		1	3.57				
596	Nordrhein-Westfalen	505450	17		1	3.57				
597	Nordrhein-Westfalen	505460	17		1	3.57				
598	Bavaria	904060	18		1	3.22				
599	Bavaria	907010	18		1	3.22				
600	Bavaria	907020	18		1	3.22				
601	Bavaria	907030	18		1	3.22				
602	Bavaria	907040	18		1	3.22				
603	Bavaria	2900347	18		1	3.22				
604	Bavaria	2900349	18		1	3.22				
605	Bavaria	2900364	18		1	3.22				
606	Bavaria	2900378	18		1	3.22				
607	Rhineland-Palatinate	701050	19		1	3.00				
608	Rhineland-Palatinate	702040	20		1	2.80				
609	Rhineland-Palatinate	702050	20		1	2.80				
610	Rhineland-Palatinate	702060	20		1	2.80				
611	Rhineland-Palatinate	702080	20		1	2.80				
612	Rhineland-Palatinate	703080	20		1	2.80				
613	Saarland	1000010	20		1	2.80				
614	Saarland	1000020	20		1	2.80				
615	Rhineland-Palatinate	2700281	20		1	2.80				
616	Rhineland-Palatinate	2700282	20		1	2.80				
617	Rhineland-Palatinate	2700283	20		1	2.80				
618	Lower Saxony	301110					0.002	1		
619	Nordrhein-Westfalen	503030					0.016	3		
620	Nordrhein-Westfalen	503190					0.034	3		
621	Nordrhein-Westfalen	504190					0.026	3		
622	Nordrhein-Westfalen	504270					0.044	4		
623	Nordrhein-Westfalen	505010					0.012	4		
624	Nordrhein-Westfalen	505050					0.02	3		
625	Baden-Württemberg	802030					0.03	4		
626	Baden-Württemberg	802040					0.002	3		
627	Baden-Württemberg	802070					0.008	3		
628	Baden-Württemberg	806010					0.04	3		
629	Baden-Württemberg	807010					0.018	3		
630	Baden-Württemberg	807020					0.02	3		
631	Baden-Württemberg	807030					0.026	3		
632	Baden-Württemberg	807050					0.016	3		
633	Baden-Württemberg	807080					0.04	3		
634	Baden-Württemberg	807100					0.042	3		
635	Baden-Württemberg	807110					0.038	3		
636	Baden-Württemberg	808030					0.004	3		
637	Baden-Württemberg	809020					0.01	3		
638	Baden-Württemberg	809030					0.036	4		
639	Bavaria	908070					0.022	4		
640	Bavaria	910010					0.05	3		
641	Bavaria	910050					0.002	3		

	A	B	C	D	E	F	G	H	I	J
1	**State**	**Region (MB)**	**Cluster GP**	**Low rate**	**High rate**	**Average cluster supply**	**Moran's I p-value**	**Moran's I q-value**	**Overlap high**	**Overlap low**
642	Bavaria	914060					0.002	3		
643	Bavaria	914090					0.026	3		
644	Bavaria	917030					0.032	3		
645	Bavaria	917050					0.004	3		
646	Bavaria	918120					0.004	3		
647	Berlin	1100000					0.002	1		
648	Brandenburg	1203010					0.002	1		
649	Brandenburg	1203020					0.002	1		
650	Brandenburg	1203030					0.002	1		
651	Brandenburg	1203050					0.006	1		
652	Brandenburg	1203070					0.01	1		
653	Brandenburg	1203080					0.022	1		
654	Brandenburg	1205010					0.038	1		
655	Brandenburg	1205020					0.002	1		
656	Saxony-Anhalt	1501010					0.008	1		
657	Saxony-Anhalt	1501040					0.018	1		
658	Saxony-Anhalt	1502060					0.004	1		
659	Saxony-Anhalt	1505010					0.008	1		
660	Saxony-Anhalt	1505020					0.006	1		
661	Saxony-Anhalt	1505030					0.002	1		
662	Thuringia	1600100					0.002	1		
663	Thuringia	1603300					0.01	1		
664	Lower Saxony	2300094					0.044	2		
665	Lower Saxony	2300433					0.03	1		
666	Lower Saxony	2300435					0.018	1		
667	Lower Saxony	2300439					0.034	1		
668	Bavaria	2900131					0.002	3		
669	Bavaria	2900140					0.014	1		
670	Bavaria	2900305					0.028	3		
671	Bavaria	2900306					0.004	1		
672	Bavaria	2900308					0.042	3		
673	Bavaria	2900355					0.002	3		
674	Bavaria	2900363					0.018	1		
675	Bavaria	2900365					0.02	1		
676	Bavaria	2900376					0.006	1		
677	Bavaria	2900381					0.034	3		
678	Saxony-Anhalt	3500160					0.006	1		
679	Thuringia	3600525					0.002	1		
