# Supplementary material for: Regional variations in multimorbidity burden among office-based physicians in Germany
Source: Eur J Public Health. 2023 Mar 15;33(3):389–95. doi: 10.1093/eurpub/ckad039 (PMC10234650; doi:10.1093/eurpub/ckad039)
Supplement: ckad039_Supplementary_Data [file ckad039_supplementary_data.zip › ckad039_Supplementary_Data/ejph-2022-10-om-0501-File009.pdf]

	A	B	C	D	E	F	G	H	I	J	K	L	M	N	O	P	Q	R	S	T	U	V	W	X	Y	Z
1	State	Region (KR)	Cluster ophthalmologist	Low rate	High rate	Average cluster supply	Moran's I p-value	Moran's I q-value	Overlap high	Overlap low	Cluster neurologist	Low rate	High rate	Average cluster supply	Moran's I p-value	Moran's I q-value	Overlap high	Overlap low	Cluster orthopaedic	Low rate	High rate	Average cluster supply	Moran's I p-value	Moran's I q-value	Overlap high	Overlap low
2	Saxony-Anhalt	150020	1		1	3.91					1		1	4.51					1		1	4.25	0.046	1	1	
3	Saxony-Anhalt	150840	1		1	3.91	0.002	1	1		1		1	4.51	0.002	1	1		1		1	4.25	0.002	1	1	
4	Saxony-Anhalt	150880	1		1	3.91	0.002	1	1		1		1	4.51	0.002	1	1		1		1	4.25	0.002	1	1	
5	Saxony-Anhalt	150910	1		1	3.91	0.002	1	1		1		1	4.51	0.002	1	1		1		1	4.25	0.002	1	1	
6	Thuringia	160520	1		1	3.91	0.004	1	1		1		1	4.51	0.008	1	1		1		1	4.25	0.046	1	1	
7	Thuringia	160760	1		1	3.91	0.002	1	1		1		1	4.51	0.002	1	1		1		1	4.25	0.002	1	1	
8	Thuringia	160770	1		1	3.91	0.002	1	1		1		1	4.51	0.002	1	1		1		1	4.25	0.012	1	1	
9	Saxony	340019	1		1	3.91	0.008	1	1		1		1	4.51	0.022	1	1		1		1	4.25	0.024	1	1	
10	Saxony	340021	1		1	3.91	0.018	1	1		1		1	4.51	0.03	1	1		1		1	4.25	0.028	1	1	
11	Saxony	340022	1		1	3.91	0.002	1	1		1		1	4.51	0.004	1	1		1		1	4.25	0.002	1	1	
12	Saxony	340023	1		1	3.91	0.042	1	1		1		1	4.51					1		1	4.25				
13	Saxony	340024	1		1	3.91	0.01	1	1		1		1	4.51	0.01	1	1		1		1	4.25	0.014	1	1	
14	Saxony	340026	1		1	3.91	0.012	1	1		1		1	4.51	0.02	1	1		1		1	4.25	0.03	1	1	
15	Saxony	340027	1		1	3.91	0.002	1	1		1		1	4.51	0.002	1	1		1		1	4.25	0.002	1	1	
16	Saxony	340028	1		1	3.91	0.004	1	1		1		1	4.51	0.008	1	1		1		1	4.25	0.014	1	1	
17	Saxony	340033	1		1	3.91	0.002	1	1		1		1	4.51	0.002	1	1		1		1	4.25	0.002	1	1	
18	Saxony	340035	1		1	3.91	0.004	1	1		1		1	4.51	0.004	1	1		1		1	4.25	0.004	1	1	
19	Saxony	340036	1		1	3.91	0.006	1	1		1		1	4.51	0.01	1	1		1		1	4.25	0.004	1	1	
20	Saxony	340037	1		1	3.91	0.004	1	1		1		1	4.51	0.022	1	1		1		1	4.25	0.014	1	1	
21	Saxony	340038	1		1	3.91	0.022	1	1		1		1	4.51					1		1	4.25	0.04	1	1	
22	Saxony	340039	1		1	3.91	0.004	1	1		1		1	4.51	0.008	1	1		1		1	4.25	0.012	1	1	
23	Saxony	340061	1		1	3.91	0.002	1	1		1		1	4.51	0.002	1	1		1		1	4.25	0.004	1	1	
24	Saxony	340081	1		1	3.91	0.008	1	1		1		1	4.51	0.026	1	1		1		1	4.25	0.012	1	1	
25	Saxony	340082	1		1	3.91	0.002	1	1		1		1	4.51	0.014	1	1		1		1	4.25	0.004	1	1	
26	Saxony-Anhalt	350017	1		1	3.91	0.002	1	1		1		1	4.51	0.002	1	1		1		1	4.25	0.004	1	1	
27	Saxony-Anhalt	350018	1		1	3.91	0.026	1	1		1		1	4.51					1		1	4.25	0.024	1	1	
28	Brandenburg	120520	2		1	4.04					1		1	4.51					1		1	4.25				
29	Brandenburg	120540	2		1	4.04					1		1	4.51					1		1	4.25				
30	Brandenburg	120610	2		1	4.04	0.004	1	1		1		1	4.51	0.006	1	1		1		1	4.25	0.002	1	1	
31	Brandenburg	120670	2		1	4.04	0.008	1	1		1		1	4.51	0.002	1	1		1		1	4.25	0.01	1	1	
32	Brandenburg	120690	2		1	4.04	0.004	1	1		1		1	4.51	0.002	1	1		1		1	4.25	0.008	1	1	
33	Brandenburg	120710	2		1	4.04	0.002	1	1		1		1	4.51	0.002	1	1		1		1	4.25	0.002	1	1	
34	Brandenburg	120720	2		1	4.04	0.01	1	1		1		1	4.51	0.014	1	1		1		1	4.25	0.038	1	1	
35	Brandenburg	120620					0.004	1			1		1	4.51	0.024	1	1		1		1	4.25	0.016	1	1	
36	Brandenburg	120660					0.002	1			1		1	4.51	0.002	2			1		1	4.25	0.004	1	1	
37	Saxony	340020					0.012	1			1		1	4.51	0.05	1	1		1		1	4.25	0.018	1	1	
38	Saxony	340029					0.002	1			1		1	4.51	0.004	1	1		1		1	4.25	0.002	1	1	
39	Saxony	340031					0.008	1			1		1	4.51	0.008	1	1		1		1	4.25	0.016	1	1	
40	Saxony	340032					0.028	1			1		1	4.51	0.05	1	1		1		1	4.25	0.034	1	1	
41	Saxony	340041					0.004	1			1		1	4.51	0.004	1	1		1		1	4.25	0.006	1	1	
42	Saxony	340042					0.006	1			1		1	4.51	0.004	1	1		1		1	4.25	0.006	1	1	
43	Saxony	340043					0.032	1			1		1	4.51	0.022	1	1		1		1	4.25	0.038	1	1	
44	Bavaria	93740	4	1		4.05					4	1		4.25					1		1	4.25				
45	Bavaria	93770	4	1		4.05					4	1		4.25					1		1	4.25				
46	Bavaria	94760	1		1	3.91	0.02	1	1		5		1	4.11	0.02	1	1		1		1	4.25	0.012	1	1	
47	Thuringia	160710	1		1	3.91	0.002	1	1		5		1	4.11	0.004	1	1		1		1	4.25	0.01	1	1	
48	Thuringia	160730	1		1	3.91	0.002	1	1		5		1	4.11	0.002	1	1		1		1	4.25	0.004	1	1	
49	Bavaria	94750	1		1	3.91													1		1	4.25	0.042	1	1	
50	Saxony-Anhalt	150870	1		1	3.91	0.002	1	1						0.008	1			1		1	4.25	0.002	1	1	
51	Saxony-Anhalt	150890	1		1	3.91	0.002	1	1						0.002	1			1		1	4.25	0.002	1	1	
52	Thuringia	160530	1		1	3.91													1		1	4.25				
53	Thuringia	160740	1		1	3.91	0.002	1	1						0.002	1			1		1	4.25	0.008	1	1	
54	Thuringia	160750	1		1	3.91	0.002	1	1						0.008	1			1		1	4.25	0.014	1	1	
55	Saxony	340083	1		1	3.91	0.008	1	1						0.026	1			1		1	4.25				
56	Bavaria	94790	4	1		4.05													1		1	4.25				
57	Bavaria	94770																	1		1	4.25				
58	Baden-Württemberg	81110	3	1		3.90	0.002	3		1	2	1		4.23	0.002	3		1	2	1		4.16	0.002	3		1
59	Baden-Württemberg	81150	3	1		3.90	0.002	3		1	2	1		4.23	0.002	3		1	2	1		4.16	0.002	3		1
60	Baden-Württemberg	81160	3	1		3.90	0.002	3		1	2	1		4.23	0.002	3		1	2	1		4.16	0.002	3		1
61	Baden-Württemberg	81170	3	1		3.90	0.002	3		1	2	1		4.23	0.002	3		1	2	1		4.16	0.002	3		1
62	Baden-Württemberg	81180	3	1		3.90	0.008	3		1	2	1		4.23	0.008	3		1	2	1		4.16	0.004	3		1
63	Baden-Württemberg	81190	3	1		3.90	0.002	3		1	2	1		4.23	0.002	3		1	2	1		4.16	0.002	3		1
64	Baden-Württemberg	81270	3	1		3.90	0.024	3		1	2	1		4.23	0.02	3		1	2	1		4.16	0.008	3		1
65	Baden-Württemberg	81350	3	1		3.90	0.01	3		1	2	1		4.23	0.002	3		1	2	1		4.16	0.036	3		1
66	Baden-Württemberg	81360	3	1		3.90	0.016	3		1	2	1		4.23	0.01	3		1	2	1		4.16	0.01	3		1
67	Baden-Württemberg	82350	3	1		3.90	0.016	3		1	2	1		4.23	0.022	3		1	2	1		4.16	0.008	3		1
68	Baden-Württemberg	82370	3	1		3.90	0.002	3		1	2	1		4.23	0.002	3		1	2	1		4.16	0.004	3		1
69	Baden-Württemberg	83250	3	1		3.90	0.012	3		1	2	1		4.23	0.004	3		1	2	1		4.16	0.004	3		1

	A	B	C	D	E	F	G	H	I	J	K	L	M	N	O	P	Q	R	S	T	U	V	W	X	Y	Z
1	State	Region (KR)	Cluster ophthalmologist	Low rate	High rate	Average cluster supply	Moran's I p-value	Moran's I q-value	Overlap high	Overlap low	Cluster neurologist	Low rate	High rate	Average cluster supply	Moran's I p-value	Moran's I q-value	Overlap high	Overlap low	Cluster orthopaedic	Low rate	High rate	Average cluster supply	Moran's I p-value	Moran's I q-value	Overlap high	Overlap low
70	Baden-Württemberg	83260	3	1		3.90	0.012	3		1	2	1		4.23	0.004	3		1	2	1		4.16	0.004	3		1
71	Baden-Württemberg	83270	3	1		3.90	0.012	3		1	2	1		4.23	0.002	3		1	2	1		4.16	0.004	3		1
72	Baden-Württemberg	83350	3	1		3.90	0.026	3		1	2	1		4.23	0.01	3		1	2	1		4.16	0.01	3		1
73	Baden-Württemberg	84150	3	1		3.90	0.002	3		1	2	1		4.23	0.002	3		1	2	1		4.16	0.002	3		1
74	Baden-Württemberg	84160	3	1		3.90	0.002	3		1	2	1		4.23	0.002	3		1	2	1		4.16	0.002	3		1
75	Baden-Württemberg	84170	3	1		3.90	0.002	3		1	2	1		4.23	0.002	3		1	2	1		4.16	0.002	3		1
76	Baden-Württemberg	84210	3	1		3.90					2	1		4.23	0.04	3		1	2	1		4.16				
77	Baden-Württemberg	84250	3	1		3.90	0.002	3		1	2	1		4.23	0.002	3		1	2	1		4.16	0.006	3		1
78	Baden-Württemberg	84260	3	1		3.90	0.004	3		1	2	1		4.23	0.002	3		1	2	1		4.16	0.012	3		1
79	Baden-Württemberg	84350	3	1		3.90	0.046	3		1	2	1		4.23	0.002	3		1	2	1		4.16	0.022	3		1
80	Baden-Württemberg	84360	3	1		3.90	0.004	3		1	2	1		4.23	0.002	3		1	2	1		4.16	0.028	3		1
81	Baden-Württemberg	84370	3	1		3.90	0.002	3		1	2	1		4.23	0.002	3		1	2	1		4.16	0.002	3		1
82	Bavaria	97730	3	1		3.90					2	1		4.23					2	1		4.16				
83	Bavaria	97740	3	1		3.90	0.014	3		1	2	1		4.23	0.004	3		1	2	1		4.16				
84	Bavaria	97750	3	1		3.90	0.004	3		1	2	1		4.23	0.002	3		1	2	1		4.16	0.034	3		1
85	Bavaria	97780	3	1		3.90	0.01	3		1	2	1		4.23	0.004	3		1	2	1		4.16				
86	Baden-Württemberg	81210	8	1		3.82					2	1		4.23					2	1		4.16				
87	Baden-Württemberg	81250	8	1		3.82	0.004	3		1	2	1		4.23	0.004	3		1	2	1		4.16	0.002	3		1
88	Baden-Württemberg	82310									2	1		4.23					2	1		4.16	0.04	3		1
89	Baden-Württemberg	82360					0.008	3			2	1		4.23	0.008	3		1	2	1		4.16	0.002	3		1
90	Brandenburg	120600	2		1	4.04	0.01	1	1		3		1	4.40	0.002	1	1		3		1	4.28	0.018	1	1	
91	Brandenburg	120650	2		1	4.04	0.006	1	1		3		1	4.40	0.002	1	1		3		1	4.28	0.012	1	1	
92	Brandenburg	120680	2		1	4.04	0.002	1	1		3		1	4.40	0.002	1	1		3		1	4.28	0.002	1	1	
93	Brandenburg	120700	2		1	4.04	0.03	1	1		3		1	4.40					3		1	4.28				
94	Brandenburg	120730	2		1	4.04	0.002	1	1		3		1	4.40	0.002	1	1		3		1	4.28	0.006	1	1	
95	Mecklenburg-Western F	130030	2		1	4.04					3		1	4.40					3		1	4.28				
96	Mecklenburg-Western F	130510	2		1	4.04	0.006	1	1		3		1	4.40	0.002	1	1		3		1	4.28	0.008	1	1	
97	Mecklenburg-Western F	130520	2		1	4.04	0.002	1	1		3		1	4.40	0.002	1	1		3		1	4.28	0.002	1	1	
98	Mecklenburg-Western F	130530	2		1	4.04	0.002	1	1		3		1	4.40	0.002	1	1		3		1	4.28	0.002	1	1	
99	Mecklenburg-Western F	130550	2		1	4.04	0.002	1	1		3		1	4.40	0.002	1	1		3		1	4.28	0.002	1	1	
100	Mecklenburg-Western F	130560	2		1	4.04	0.002	1	1		3		1	4.40	0.004	1	1		3		1	4.28	0.002	1	1	
101	Mecklenburg-Western F	130570	2		1	4.04	0.002	1	1		3		1	4.40	0.002	1	1		3		1	4.28	0.002	1	1	
102	Mecklenburg-Western F	130580	2		1	4.04	0.02	1	1		3		1	4.40	0.014	1	1		3		1	4.28	0.046	1	1	
103	Mecklenburg-Western F	130590	2		1	4.04	0.002	1	1		3		1	4.40	0.002	1	1		3		1	4.28	0.002	1	1	
104	Mecklenburg-Western F	130600	2		1	4.04	0.002	1	1		3		1	4.40	0.004	1	1		3		1	4.28	0.004	1	1	
105	Mecklenburg-Western F	130610	2		1	4.04					3		1	4.40					3		1	4.28				
106	Mecklenburg-Western F	130620	2		1	4.04	0.002	1	1		3		1	4.40	0.002	1	1		3		1	4.28	0.004	1	1	
107	Mecklenburg-Western F	130540	2		1	4.04													3		1	4.28				
108	Schleswig-Holstein	10510	5	1		3.94					7	1		4.20					4	1		4.36				
109	Schleswig-Holstein	10560	5	1		3.94					7	1		4.20					4	1		4.36	0.008	3		1
110	Schleswig-Holstein	10580	5	1		3.94					7	1		4.20					4	1		4.36	0.032	3		1
111	Schleswig-Holstein	10600	5	1		3.94	0.02	3		1	7	1		4.20					4	1		4.36	0.004	3		1
112	Schleswig-Holstein	10610	5	1		3.94					7	1		4.20					4	1		4.36	0.014	3		1
113	Schleswig-Holstein	10620	5	1		3.94					7	1		4.20					4	1		4.36	0.02	3		1
114	Hamburg	20000	5	1		3.94	0.038	3		1	7	1		4.20					4	1		4.36	0.004	3		1
115	Lower Saxony	33520	5	1		3.94					7	1		4.20	0.028	3		1	4	1		4.36				
116	Lower Saxony	33530	5	1		3.94	0.03	3		1	7	1		4.20	0.04	3		1	4	1		4.36	0.012	3		1
117	Lower Saxony	33570	5	1		3.94					7	1		4.20					4	1		4.36	0.048	3		1
118	Lower Saxony	33590	5	1		3.94	0.048	3		1	7	1		4.20					4	1		4.36	0.004	3		1
119	Bremen	40120	5	1		3.94					7	1		4.20					4	1		4.36				
120	Schleswig-Holstein	10020	5	1		3.94													4	1		4.36				
121	Schleswig-Holstein	10570	5	1		3.94													4	1		4.36	0.022	3		1
122	Bavaria	91790	3	1		3.90	0.008	3		1	4	1		4.25	0.008	3		1	5	1		4.75	0.006	3		1
123	Bavaria	91810	3	1		3.90	0.008	3		1	4	1		4.25	0.01	3		1	5	1		4.75	0.008	3		1
124	Bavaria	91880	3	1		3.90	0.01	3		1	4	1		4.25	0.014	3		1	5	1		4.75	0.002	3		1
125	Bavaria	91900	3	1		3.90					4	1		4.25	0.02	3		1	5	1		4.75	0.008	3		1
126	Bavaria	97710	3	1		3.90	0.002	3		1	4	1		4.25	0.014	3		1	5	1		4.75	0.01	3		1
127	Bavaria	91620	4	1		4.05	0.004	3		1	4	1		4.25	0.02	3		1	5	1		4.75	0.004	3		1
128	Bavaria	91740	4	1		4.05	0.002	3		1	4	1		4.25	0.018	3		1	5	1		4.75	0.002	3		1
129	Bavaria	91750	4	1		4.05	0.022	3		1	4	1		4.25					5	1		4.75	0.03	3		1
130	Bavaria	91770	4	1		4.05	0.01	3		1	4	1		4.25					5	1		4.75	0.032	3		1
131	Bavaria	91780	4	1		4.05	0.004	3		1	4	1		4.25	0.026	3		1	5	1		4.75	0.016	3		1
132	Bavaria	91830	4	1		4.05	0.024	3		1	4	1		4.25					5	1		4.75	0.01	3		1
133	Bavaria	91840	4	1		4.05	0.002	3		1	4	1		4.25	0.004	3		1	5	1		4.75	0.002	3		1
134	Bavaria	91860	4	1		4.05	0.002	3		1	4	1		4.25	0.004	3		1	5	1		4.75	0.006	3		1
135	Bavaria	91870	4	1		4.05	0.018	3		1	4	1		4.25					5	1		4.75	0.004	3		1
136	Bavaria	91730					0.05	3			4	1		4.25	0.02	3		1	5	1		4.75	0.002	3		1
137	Bavaria	91820									4	1		4.25					5	1		4.75	0.038	3		1

	A	B	C	D	E	F	G	H	I	J	K	L	M	N	O	P	Q	R	S	T	U	V	W	X	Y	Z
1	State	Region (KR)	Cluster ophthalmologist	Low rate	High rate	Average cluster supply	Moran's I p-value	Moran's I q-value	Overlap high	Overlap low	Cluster neurologist	Low rate	High rate	Average cluster supply	Moran's I p-value	Moran's I q-value	Overlap high	Overlap low	Cluster orthopaedic	Low rate	High rate	Average cluster supply	Moran's I p-value	Moran's I q-value	Overlap high	Overlap low
138	Thuringia	160510	1		1	3.91	0.01	1	1		5		1	4.11	0.012	1	1		6		1	4.33				
139	Thuringia	160610	1		1	3.91	0.004	1	1		5		1	4.11					6		1	4.33	0.002	1	1	
140	Thuringia	160640	1		1	3.91	0.004	1	1		5		1	4.11	0.01	1	1		6		1	4.33	0.006	1	1	
141	Thuringia	160660	1		1	3.91					5		1	4.11	0.04	1	1		6		1	4.33				
142	Thuringia	160670	1		1	3.91	0.002	1	1		5		1	4.11	0.004	1	1		6		1	4.33	0.026	1	1	
143	Thuringia	160680	1		1	3.91	0.002	1	1		5		1	4.11	0.004	1	1		6		1	4.33	0.008	1	1	
144	Thuringia	160690	1		1	3.91	0.03	1	1		5		1	4.11	0.006	1	1		6		1	4.33	0.044	1	1	
145	Thuringia	160700	1		1	3.91	0.002	1	1		5		1	4.11	0.004	1	1		6		1	4.33	0.012	1	1	
146	Hessen	65350									5		1	4.11					6		1	4.33				
147	Hessen	66310									5		1	4.11					6		1	4.33				
148	Hessen	66320									5		1	4.11					6		1	4.33				
149	Hessen	66360									5		1	4.11					6		1	4.33				
150	Bavaria	96720									5		1	4.11					6		1	4.33				
151	Bavaria	96730									5		1	4.11					6		1	4.33				
152	Thuringia	160630					0.03	1			5		1	4.11	0.016	1	1		6		1	4.33	0.034	1	1	
153	Hessen	65320	9	1		3.43					10	1		4.00					6		1	4.33				
154	Hessen	65340	9	1		3.43					10	1		4.00					6		1	4.33				
155	Thuringia	160620	1		1	3.91	0.002	1	1						0.016	1			6		1	4.33	0.002	1	1	
156	Thuringia	160650	1		1	3.91	0.002	1	1						0.002	1			6		1	4.33	0.002	1	1	
157	Hessen	64350	8	1		3.82													6		1	4.33				
158	Hessen	64400	8	1		3.82													6		1	4.33				
159	Lower Saxony	31550	11		1	3.77													6		1	4.33				
160	Lower Saxony	31520																	6		1	4.33	0.036	1	1	
161	Lower Saxony	31560																	6		1	4.33	0.04	1	1	
162	Nordrhein-Westfalen	57620																	6		1	4.33				
163	Hessen	65310																	6		1	4.33				
164	Hessen	66110																	6		1	4.33				
165	Hessen	66330																	6		1	4.33				
166	Hessen	66340																	6		1	4.33				
167	Hessen	66350																	6		1	4.33				
168	Saarland	100410	7	1		4.00	0.034	3		1	6	1		4.40					7	1		4.60	0.016	3		1
169	Saarland	100420	7	1		4.00					6	1		4.40					7	1		4.60	0.02	3		1
170	Saarland	100430	7	1		4.00	0.038	3		1	6	1		4.40					7	1		4.60	0.034	3		1
171	Saarland	100440	7	1		4.00	0.006	3		1	6	1		4.40	0.002	3		1	7	1		4.60	0.002	3		1
172	Saarland	100460	7	1		4.00					6	1		4.40					7	1		4.60				
173	Lower Saxony	34540	6	1		4.00					7	1		4.20					8	1		4.20				
174	Lower Saxony	34590	6	1		4.00	0.006	3		1	7	1		4.20					8	1		4.20	0.024	3		1
175	Lower Saxony	34600	6	1		4.00					7	1		4.20					8	1		4.20				
176	Lower Saxony	32560	11		1	3.77					7	1		4.20					8	1		4.20				
177	Nordrhein-Westfalen	57700	11		1	3.77					7	1		4.20					8	1		4.20				
178	Lower Saxony	32510									7	1		4.20	0.038	3		1	8	1		4.20	0.048	3		1
179	Lower Saxony	34530									7	1		4.20					8	1		4.20				
180	Lower Saxony	34580									7	1		4.20					8	1		4.20				
181	Nordrhein-Westfalen	55150	6	1		4.00	0.04	3		1	9	1		4.09					8	1		4.20				
182	Nordrhein-Westfalen	55540	6	1		4.00					9	1		4.09					8	1		4.20				
183	Nordrhein-Westfalen	55580	6	1		4.00	0.014	3		1	9	1		4.09					8	1		4.20				
184	Nordrhein-Westfalen	55620	6	1		4.00					9	1		4.09					8	1		4.20				
185	Nordrhein-Westfalen	59780									12		1	4.60					8	1		4.20				
186	Nordrhein-Westfalen	57740									13	1		4.00					8	1		4.20				
187	Lower Saxony	34040	6	1		4.00													8	1		4.20				
188	Lower Saxony	34560	6	1		4.00	0.044	3		1									8	1		4.20				
189	Nordrhein-Westfalen	55660	6	1		4.00	0.002	3		1									8	1		4.20	0.024	3		1
190	Nordrhein-Westfalen	55700	6	1		4.00	0.01	3		1									8	1		4.20				
191	Nordrhein-Westfalen	57540	6	1		4.00													8	1		4.20				
192	Nordrhein-Westfalen	59150	6	1		4.00													8	1		4.20				
193	Lower Saxony	32570	11		1	3.77													8	1		4.20				
194	Nordrhein-Westfalen	57110																	8	1		4.20				
195	Nordrhein-Westfalen	57580																	8	1		4.20				
196	Nordrhein-Westfalen	57660																	8	1		4.20				
197	Nordrhein-Westfalen	59740																	8	1		4.20				
198	Hessen	64110	8	1		3.82					8	1		3.92					9	1		4.60				
199	Hessen	64330	8	1		3.82					8	1		3.92					9	1		4.60	0.036	3		1
200	Hessen	64360	8	1		3.82					8	1		3.92					9	1		4.60				
201	Rhineland-Palatinate	73150	8	1		3.82					8	1		3.92					9	1		4.60				
202	Hessen	64320	8	1		3.82													9	1		4.60				
203	Nordrhein-Westfalen	53580									11	1		4.12					10		1	4.00				
204	Nordrhein-Westfalen	53341																	10		1	4.00				
205	Nordrhein-Westfalen	53342																	10		1	4.00				

	A	B	C	D	E	F	G	H	I	J	K	L	M	N	O	P	Q	R	S	T	U	V	W	X	Y	Z
1	State	Region (KR)	Cluster ophthalmologist	Low rate	High rate	Average cluster supply	Moran's I p-value	Moran's I q-value	Overlap high	Overlap low	Cluster neurologist	Low rate	High rate	Average cluster supply	Moran's I p-value	Moran's I q-value	Overlap high	Overlap low	Cluster orthopaedic	Low rate	High rate	Average cluster supply	Moran's I p-value	Moran's I q-value	Overlap high	Overlap low
206	Nordrhein-Westfalen	53660																	10		1	4.00				
207	Nordrhein-Westfalen	53700																	10		1	4.00				
208	Nordrhein-Westfalen	51110	10	1		4.00					11	1		4.12					11	1		4.00				
209	Nordrhein-Westfalen	53740	9	1		3.43					10	1		4.00					12	1		4.17				
210	Nordrhein-Westfalen	59580	9	1		3.43					10	1		4.00					12	1		4.17				
211	Nordrhein-Westfalen	59660	9	1		3.43					10	1		4.00					12	1		4.17				
212	Nordrhein-Westfalen	59700	9	1		3.43					10	1		4.00					12	1		4.17				
213	Rhineland-Palatinate	71320	9	1		3.43					10	1		4.00					12	1		4.17				
214	Nordrhein-Westfalen	59620									12		1	4.60					12	1		4.17				
215	Nordrhein-Westfalen	55130									9	1		4.09					13		1	4.40				
216	Nordrhein-Westfalen	59110	12		1	4.00					12		1	4.60					13		1	4.40				
217	Nordrhein-Westfalen	51130									12		1	4.60					13		1	4.40				
218	Nordrhein-Westfalen	59160									12		1	4.60					13		1	4.40				
219	Nordrhein-Westfalen	59540									12		1	4.60					13		1	4.40				
220	Lower Saxony	32520	11		1	3.77													14		1	4.00				
221	Nordrhein-Westfalen	51700									9	1		4.09					15	1		4.00				
222	Nordrhein-Westfalen	51190																	15	1		4.00				
223	Nordrhein-Westfalen	51120	14		1	4.00					14		1	4.00					16		1	4.50				
224	Nordrhein-Westfalen	51170																	16		1	4.50				
225	Nordrhein-Westfalen	53820																	17		1	4.40				
226	Rhineland-Palatinate	71110																	17		1	4.40				
227	Rhineland-Palatinate	71370																	17		1	4.40				
228	Rhineland-Palatinate	71380																	17		1	4.40				
229	Rhineland-Palatinate	71430																	17		1	4.40				
230	Nordrhein-Westfalen	53140									11	1		4.12					18	1		5.00				
231	Brandenburg	120640	2		1	4.04	0.034	1	1		3		1	4.40	0.028	1	1						0.038	1		
232	Bavaria	97610	3	1		3.90					4	1		4.25												
233	Bavaria	91610	4	1		4.05	0.004	3		1	4	1		4.25	0.016	3		1								
234	Bavaria	91710	4	1		4.05					4	1		4.25												
235	Bavaria	91720	4	1		4.05					4	1		4.25									0.036	4		
236	Bavaria	91760	4	1		4.05	0.006	3		1	4	1		4.25												
237	Bavaria	91850	4	1		4.05	0.018	3		1	4	1		4.25	0.04	3		1								
238	Bavaria	91890	4	1		4.05					4	1		4.25												
239	Bavaria	92710	4	1		4.05					4	1		4.25												
240	Bavaria	92720	4	1		4.05					4	1		4.25												
241	Bavaria	92730	4	1		4.05	0.004	3		1	4	1		4.25												
242	Bavaria	92740	4	1		4.05	0.016	3		1	4	1		4.25												
243	Bavaria	92750	4	1		4.05					4	1		4.25												
244	Bavaria	92760	4	1		4.05					4	1		4.25												
245	Bavaria	92770	4	1		4.05					4	1		4.25												
246	Bavaria	92780	4	1		4.05					4	1		4.25												
247	Bavaria	92790	4	1		4.05					4	1		4.25												
248	Bavaria	93620	4	1		4.05					4	1		4.25												
249	Bavaria	93710	4	1		4.05					4	1		4.25												
250	Bavaria	93720	4	1		4.05					4	1		4.25												
251	Bavaria	93730	4	1		4.05	0.02	3		1	4	1		4.25												
252	Bavaria	93750	4	1		4.05	0.048	3		1	4	1		4.25												
253	Bavaria	93760	4	1		4.05					4	1		4.25												
254	Bavaria	95740	4	1		4.05					4	1		4.25												
255	Bavaria	95760	4	1		4.05					4	1		4.25												
256	Bavaria	95770									4	1		4.25												
257	Thuringia	160720	1		1	3.91	0.03	1	1		5		1	4.11	0.004	1	1						0.016	1		
258	Bavaria	94780	13	1		4.00					5		1	4.11												
259	Bavaria	96740	13	1		4.00					5		1	4.11												
260	Bavaria	94730									5		1	4.11	0.038	1	1									
261	Bavaria	96780									5		1	4.11												
262	Lower Saxony	33580	11		1	3.77					7	1		4.20												
263	Schleswig-Holstein	10530									7	1		4.20												
264	Schleswig-Holstein	10540									7	1		4.20												
265	Lower Saxony	33550									7	1		4.20												
266	Lower Saxony	33560									7	1		4.20	0.02	3		1								
267	Lower Saxony	33610									7	1		4.20												
268	Lower Saxony	34030									7	1		4.20												
269	Lower Saxony	34510									7	1		4.20												
270	Lower Saxony	34520									7	1		4.20												
271	Lower Saxony	34550									7	1		4.20												
272	Lower Saxony	34570									7	1		4.20												
273	Lower Saxony	34610									7	1		4.20												

	A	B	C	D	E	F	G	H	I	J	K	L	M	N	O	P	Q	R	S	T	U	V	W	X	Y	Z
1	**State**	**Region (KR)**	**Cluster ophthalmologist**	**Low rate**	**High rate**	**Average cluster supply**	**Moran's I p-value**	**Moran's I q-value**	**Overlap high**	**Overlap low**	**Cluster neurologist**	**Low rate**	**High rate**	**Average cluster supply**	**Moran's I p-value**	**Moran's I q-value**	**Overlap high**	**Overlap low**	**Cluster orthopaedic**	**Low rate**	**High rate**	**Average cluster supply**	**Moran's I p-value**	**Moran's I q-value**	**Overlap high**	**Overlap low**
274	Lower Saxony	34620									7	1		4.20												
275	Bremen	40110									7	1		4.20												
276	Hessen	64140	8	1		3.82	0.05	3		1	8	1		3.92	0.048	3		1					0.042	4		
277	Rhineland-Palatinate	73310	8	1		3.82					8	1		3.92												
278	Rhineland-Palatinate	73390	8	1		3.82					8	1		3.92												
279	Hessen	64390									8	1		3.92												
280	Nordrhein-Westfalen	55120									9	1		4.09												
281	Nordrhein-Westfalen	51160									11	1		4.12												
282	Nordrhein-Westfalen	51620									11	1		4.12												
283	Nordrhein-Westfalen	53150									11	1		4.12												
284	Nordrhein-Westfalen	53160									11	1		4.12												
285	Nordrhein-Westfalen	53620									11	1		4.12												
286	Nordrhein-Westfalen	53780									11	1		4.12												
287	Nordrhein-Westfalen	51200									12		1	4.60												
288	Nordrhein-Westfalen	51220									12		1	4.60												
289	Nordrhein-Westfalen	51240									12		1	4.60												
290	Nordrhein-Westfalen	59130									12		1	4.60												
291	Nordrhein-Westfalen	59140									12		1	4.60												
292	Nordrhein-Westfalen	51140	14		1	4.00					14		1	4.00												
293	Nordrhein-Westfalen	51660	14		1	4.00					14		1	4.00												
294	Saxony-Anhalt	150030	1		1	3.91	0.012	1	1														0.014	1		
295	Saxony-Anhalt	150850	1		1	3.91	0.002	1	1														0.002	1		
296	Brandenburg	120630	2		1	4.04	0.004	1	1						0.01	1							0.04	1		
297	Saxony-Anhalt	150860	2		1	4.04	0.002	1	1						0.006	1							0.002	1		
298	Saxony-Anhalt	150900	2		1	4.04	0.004	1	1						0.034	1							0.018	1		
299	Berlin	310016	2		1	4.04	0.002	1	1						0.002	1							0.002	1		
300	Bavaria	97720	3	1		3.90	0.02	3		1					0.018	3										
301	Bavaria	97760	3	1		3.90																				
302	Bavaria	97770	3	1		3.90	0.028	3		1					0.008	3										
303	Bavaria	97790	3	1		3.90	0.002	3		1					0.008	3							0.006	4		
304	Bavaria	97800	3	1		3.90									0.016	3										
305	Bavaria	94720	4	1		4.05																				
306	Hessen	64120	8	1		3.82																				
307	Hessen	64130	8	1		3.82																				
308	Hessen	64310	8	1		3.82																				
309	Hessen	64340	8	1		3.82																				
310	Hessen	64370	8	1		3.82																				
311	Hessen	64380	8	1		3.82																				
312	Rhineland-Palatinate	73140	8	1		3.82																				
313	Rhineland-Palatinate	73320	8	1		3.82																				
314	Rhineland-Palatinate	73380	8	1		3.82																				
315	Baden-Württemberg	81260	8	1		3.82	0.018	3		1													0.016	3		
316	Baden-Württemberg	81280	8	1		3.82	0.016	3		1					0.038	3							0.04	3		
317	Baden-Württemberg	82150	8	1		3.82																	0.036	3		
318	Baden-Württemberg	82210	8	1		3.82																				
319	Baden-Württemberg	82220	8	1		3.82																				
320	Baden-Württemberg	82250	8	1		3.82																				
321	Baden-Württemberg	82260	8	1		3.82																				
322	Bavaria	96630	8	1		3.82																				
323	Bavaria	96710	8	1		3.82																				
324	Bavaria	96760	8	1		3.82																				
325	Bavaria	96770	8	1		3.82																				
326	Bavaria	96790	8	1		3.82																				
327	Lower Saxony	31010	11		1	3.77																				
328	Lower Saxony	31020	11		1	3.77																				
329	Lower Saxony	31030	11		1	3.77																				
330	Lower Saxony	31510	11		1	3.77																				
331	Lower Saxony	31530	11		1	3.77	0.034	1	1														0.024	1		
332	Lower Saxony	31570	11		1	3.77																				
333	Lower Saxony	31580	11		1	3.77																				
334	Lower Saxony	32411	11		1	3.77																				
335	Lower Saxony	32412	11		1	3.77																				
336	Lower Saxony	32540	11		1	3.77																				
337	Lower Saxony	32550	11		1	3.77																				
338	Lower Saxony	33510	11		1	3.77																				
339	Bavaria	94710	13	1		4.00																				
340	Bavaria	94740	13	1		4.00																				
341	Bavaria	95720	13	1		4.00																				

	A	B	C	D	E	F	G	H	I	J	K	L	M	N	O	P	Q	R	S	T	U	V	W	X	Y	Z
1	**State**	**Region (KR)**	**Cluster ophthalmologist**	**Low rate**	**High rate**	**Average cluster supply**	**Moran's I p-value**	**Moran's I q-value**	**Overlap high**	**Overlap low**	**Cluster neurologist**	**Low rate**	**High rate**	**Average cluster supply**	**Moran's I p-value**	**Moran's I q-value**	**Overlap high**	**Overlap low**	**Cluster orthopaedic**	**Low rate**	**High rate**	**Average cluster supply**	**Moran's I p-value**	**Moran's I q-value**	**Overlap high**	**Overlap low**
342	Schleswig-Holstein	10550																					0.044	3		
343	Lower Saxony	33540					0.046	1																		
344	Baden-Württemberg	82160													0.028	4							0.022	3		
345	Bavaria	91800																					0.04	3		
346	Saxony-Anhalt	150830					0.004	1							0.042	1							0.004	1		
