# Supplementary figures and images for: Regional variations in multimorbidity burden among office-based physicians in Germany
Source: Eur J Public Health. 2023 Mar 15;33(3):389–95. doi: 10.1093/eurpub/ckad039 (PMC10234650; doi:10.1093/eurpub/ckad039)

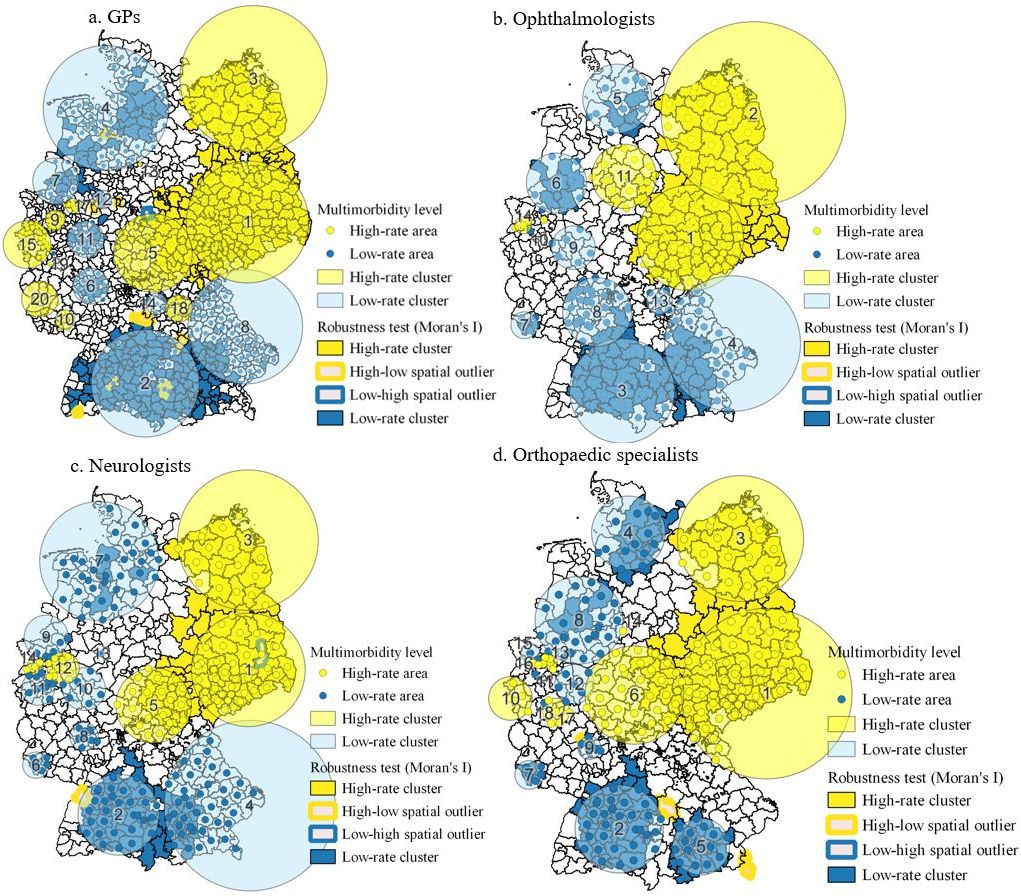

Supplement: ckad039_Supplementary_Data [file ckad039_supplementary_data.zip › ckad039_Supplementary_Data/ejph-2022-10-om-0501-File010.jpg]

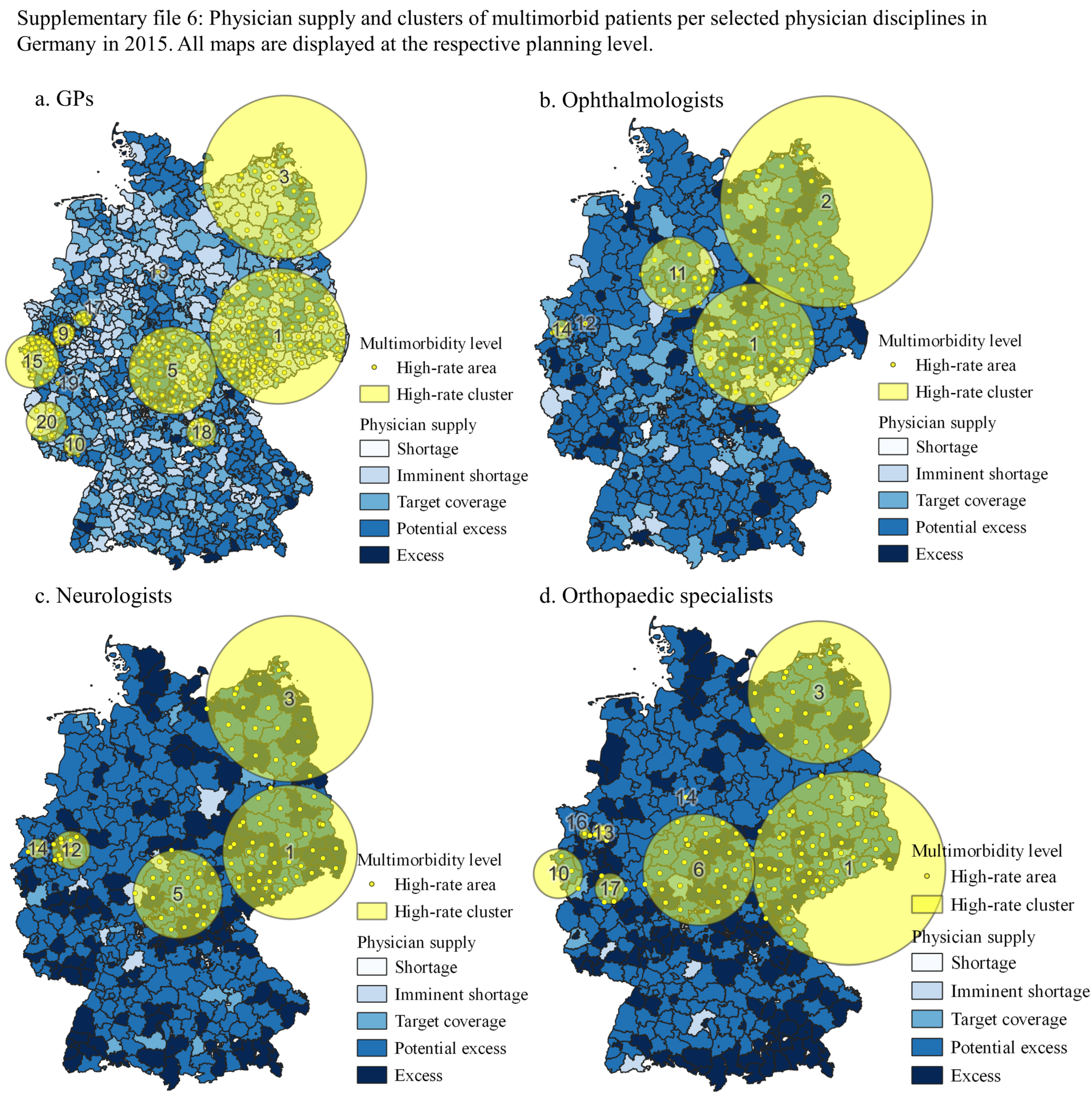

Supplement: ckad039_Supplementary_Data [file ckad039_supplementary_data.zip › ckad039_Supplementary_Data/ejph-2022-10-om-0501-File011.tif]
